# Detecting local diversity‐dependence in diversification

**DOI:** 10.1111/evo.13482

**Published:** 2018-04-24

**Authors:** Liang Xu, Rampal S. Etienne

**Affiliations:** ^1^ Groningen Institute for Evolutionary Life Sciences University of Groningen PO Box 11103 Groningen 9700 CC The Netherlands

**Keywords:** Diversity‐dependence, macroevolution, parametric bootstrap, phylogeny, simulations

## Abstract

Whether there are ecological limits to species diversification is a hotly debated topic. Molecular phylogenies show slowdowns in lineage accumulation, suggesting that speciation rates decline with increasing diversity. A maximum‐likelihood (ML) method to detect diversity‐dependent (DD) diversification from phylogenetic branching times exists, but it assumes that diversity‐dependence is a global phenomenon and therefore ignores that the underlying species interactions are mostly local, and not all species in the phylogeny co‐occur locally. Here, we explore whether this ML method based on the nonspatial diversity‐dependence model can detect local diversity‐dependence, by applying it to phylogenies, simulated with a spatial stochastic model of local DD speciation, extinction, and dispersal between two local communities. We find that type I errors (falsely detecting diversity‐dependence) are low, and the power to detect diversity‐dependence is high when dispersal rates are not too low. Interestingly, when dispersal is high the power to detect diversity‐dependence is even higher than in the nonspatial model. Moreover, estimates of intrinsic speciation rate, extinction rate, and ecological limit strongly depend on dispersal rate. We conclude that the nonspatial DD approach can be used to detect diversity‐dependence in clades of species that live in not too disconnected areas, but parameter estimates must be interpreted cautiously.

Understanding the potential ecological limits to species diversification remains a hotly debated topic (Harmon and Harrison 2015; Rabosky and Hurlbert 2015; Kozak and Wiens 2016). The rising availability of molecular data to create phylogenies has motivated the development of a variety of methods to interpret lineage diversification and better understand its mechanisms. Such methods include the lineages‐through‐time (LTT) plot—a semilogarithmic plot that tracks the number of species that have descendants at the present through time. LTT plots indicate that species accumulation slows through evolutionary time (Moen and Morlon 2014). This decreasing rate of diversification has often been interpreted as a sign of diversity‐dependence (Pybus and Harvey 2000; Weir 2006; Phillimore and Price 2008; Rabosky and Lovette 2008a, 2008b), resulting in the absence of a correlation between the crown age of phylogenies and current‐day diversity. Nevertheless, other explanations also exist including time‐dependent speciation and/or extinction rates, or the protracted nature of speciation (Etienne and Rosindell 2012; Moen and Morlon 2014).

To infer the presence of diversity‐dependent (DD) diversification from molecular phylogenies containing only extant taxa, the standard procedure is to compare the fit of a DD model (Valentine 1973; Sepkoski 1978) to a model with no diversity‐dependence, which is commonly known as the constant‐rates (CR) birth–death model (Raup et al. 1973). DD models assume that evolutionary radiations are facilitated by ecological opportunity (Schluter 2000), and that speciation is more likely to happen when diversity is low. Importantly, although extinct species leave no descendants at present, they may have affected diversification and hence also the phylogenetic patterns that are observed at present. An algorithm to compute the likelihood of a model based on this idea from a species‐level molecular phylogeny of present‐day species (which may be incomplete as long as the number of species not represented in the tree is specified) was developed a few years ago (Etienne et al. 2011). This likelihood not only allows for estimation of lineage diversification rates but can be used in likelihood‐based tests to compare the model to other diversity‐independent models. Standard tests based on the likelihood ratio and (corrected) Akaike information criterion have recently been reported to be inadequate for the comparison of DD versus CR models because of violation of some of the assumptions leading to the χ^2^ distribution used in these tests, but a bootstrap likelihood ratio test is available as an alternative (Etienne et al. 2016). In summary, we currently have the tools to check whether and when diversity‐dependence can be detected.

However, current models used to detect DD diversification on molecular phylogenies assume that the global species richness of a clade determines its rate of diversification, even if the species belonging to the clade do not interact, for example, because of disjunct spatial distributions. Hence, the question arises how we can detect diversity‐dependence in such occasions. The ideal solution would be a test with a spatial model that incorporates diversity‐dependence. In 2011, Goldberg et al. constructed a spatial model, the geographic state speciation and extinction (GeoSSE) model (Goldberg et al. 2011), which includes biogeographic states and allows state changes at speciation and through local extinction. However, it is built on the mathematical framework of the binary state speciation and extinction (BiSSE) model (Maddison et al. 2007) and thus inherits the assumption from the BiSSE model that all the evolutionary parameters are constant or time‐dependent (Rabosky and Glor 2010), but not strictly DD. Computing the likelihood for a spatial diversity‐dependence model remains a challenge, however, because it needs to keep track of all species, even currently extinct ones, in all spatial locations. An alternative solution is to test whether the above‐mentioned bootstrap likelihood ratio test based on the nonspatial diversity‐dependence model can detect local diversity‐dependence. In this article we explore this option.

We extend the DD diversification model to two locations connected by dispersal, where both speciation and dispersal are DD. In this spatial diversity‐dependence model, we incorporate both allopatric speciation and sympatric speciation and assume constant extinction because DD extinction seems at odds with empirical phylogenies (Etienne et al. 2011). We simulate phylogenetic trees following this model using various values for its parameters, to subsequently estimate parameters using a nonspatial DD model (Etienne et al. 2011). We employ the bootstrap likelihood test to explore whether we can detect diversity‐dependence when data are simulated under the spatial diversity‐dependence model.

## Materials & Methods

### MODEL

We introduce the simplest spatial DD diversification model by assuming two regions, denoted by 1 and 2. We call this model the spatial model. It is an extension of the DD diversification model of Etienne et al. (2012), which has no spatial structure, and hence will be called the nonspatial model. Our spatial model considers local macroevolutionary processes (sympatric speciation and local extinction) as well as species interactions between locations (through dispersal and allopatric speciation). Our aim is to explore whether the simpler nonspatial model can detect diversity‐dependence from simulations under the more complicated spatial model, and whether parameters estimated using the nonspatial model relate in an informative way to the true parameters of the generating spatial model.

We assume that sympatric speciation rates are linear functions of the number of species present on the locations. We denote the number of species on locations 1 and 2 by *n*_1_ and *n*_2_, respectively. Sympatric speciation rates $\lambda_{1}\left(n_{1}\right)$ and $\lambda_{2}(n_{2}$) for both locations are defined as follows:

(1) $$\begin{array}{ccc} \lambda_{1}\left(n_{1}\right) & = & \max\left(0,\lambda_{1,0}-\left(\lambda_{1,0}-\mu \right)\frac{n_{1}}{K_{1}}\right) \end{array}$$

(2) $$\begin{array}{ccc} \lambda_{2}\left(n_{2}\right) & = & \max\left(0,\lambda_{2,0}-\left(\lambda_{2,0}-\mu \right)\frac{n_{2}}{K_{2}}\right). \end{array}$$

Here, λ_1, 0_ and λ_2, 0_ are the intrinsic speciation rates of the two locations; these are the rates when diversity is 0. Furthermore, *K*_1_ and *K*_2_ can be interpreted as the carrying capacities for the two locations. We can rewrite these expressions as

(3) $$\begin{array}{ccc} \lambda_{1}\left(n_{1}\right) & = & \max\left(0,\lambda_{1,0}\left(1-\frac{n_{1}}{K_{1}^{\prime }}\right)\right) \end{array}$$

(4) $$\begin{array}{ccc} \lambda_{2}\left(n_{2}\right) & = & \max\left(0,\lambda_{2,0}\left(1-\frac{n_{2}}{K_{2}^{\prime }}\right)\right), \end{array}$$

where we have defined

(5) $$K_{i}^{\prime }=\lambda_{i,0}K_{i}/\left(\lambda_{i,0}-\mu \right).$$

The parameter $K_{i}^{\prime }$ can be interpreted as the maximum number of niches that the species in the clade can occupy (Etienne et al. 2011), and hence it is an ecological limit to diversity.

Dispersal between the two regions is also assumed to be DD:

(6) $$\begin{array}{ccc} M_{1\to 2}\left(n_{2}\right) & = & \max\left(0,M_{0}\left(1-\frac{n_{2}}{K_{2}^{\prime }}\right)\right) \end{array}$$

(7) $$\begin{array}{ccc} M_{2\to 1}\left(n_{1}\right) & = & \max\left(0,M_{0}\left(1-\frac{n_{1}}{K_{1}^{\prime }}\right)\right), \end{array}$$

where *M*_0_ is the intrinsic dispersal rate when diversity is 0 in the receiving region, and the notation a→b stands for dispersal from location *a* to location *b*. Equations 6 and 7 show that dispersal rates are dependent on the diversity of the location species are dispersing to. Diversity‐dependence is often based on a niche‐filling argument: as diversity increases, it is increasingly harder for a new species to enter the community and find its own niche to establish in the community. Entering the community can occur either through speciation or through immigration. Hence, the rate of sympatric speciation and of dispersal both depend on the diversity in the location that the new species enters.

The consequence of dispersal is that some species inhabit both regions at the same time; we will refer to these as “widespread species.” In contrast, we will call species residing on a single location “endemic species.” In our model we incorporate allopatric speciation, that is, the split of a species that is present on both locations into two species, each present on one location. The allopatric speciation rate is assumed to be negatively related to the intrinsic dispersal rate

(8) $$\lambda_{12}=\frac{\lambda_{12,0}}{M_{0}},$$

where λ_12, 0_ is the allopatric speciation rate when the dispersal rate equals unity. Equation (8) shows that as species dispersal between locations increases, allopatric speciation becomes less likely. Finally, we consider local extinction rates to be constant because empirical phylogenies suggest they do not increase with diversity, and we consider them equal for the two locations $\mu_{1,n}=\mu_{2,n}=\mu$ for simplicity.

When the widespread species goes extinct on one location, it becomes an endemic species. We call this evolutionary process “range contraction.” For widespread species, complete extinction can only occur by two consecutive local extinction events without species dispersal between these events, that is, contraction followed by local extinction. Thus we do not allow global extinction, that is, immediate complete extinction for widespread species that is in line with the GeoSSE model (Goldberg et al. 2011).

Theoretically, it is possible to compute the likelihood of our model given a phylogeny using the hidden Markov approach of Etienne et al. (2012). However, because we have to consider all the possible combinations of endemic and widespread species richness (i.e. (a,b,c) with *a* endemic species on location A, *b* endemic species on location B, and *c* widespread species), not only for the lineages in the phylogeny, but also for now‐extinct species, the state space of the model is huge leading to severe computational and numerical problems. Hence, our aim here is to explore whether the computationally manageable nonspatial model (Etienne et al. 2011) can be used for inferring diversity dependence from phylogenies simulated under the spatial model.

### SIMULATION

**Figure 1:**
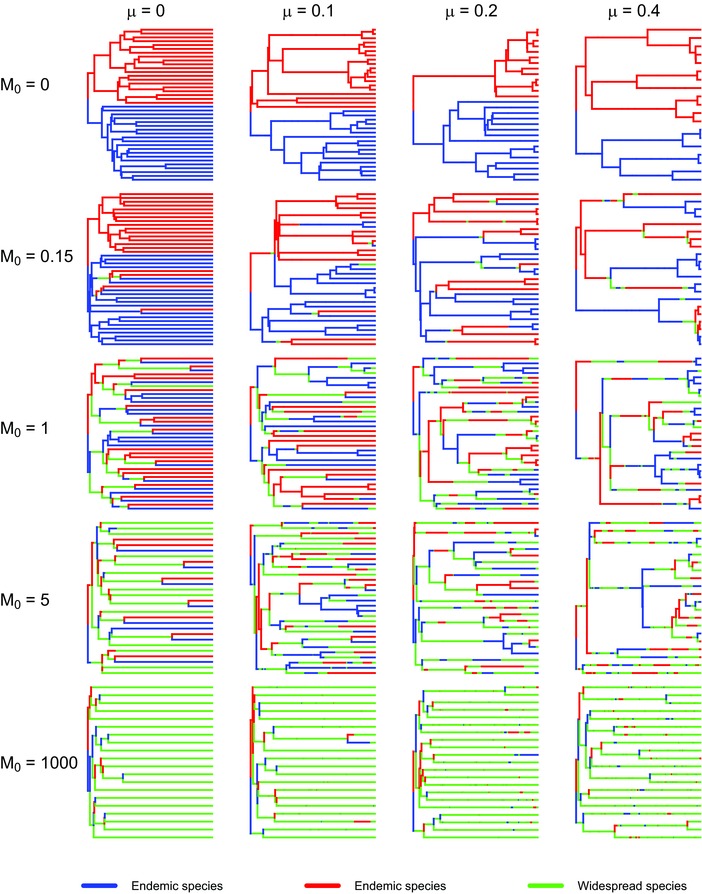
Examples of phylogenetic trees produced in Scenario 1. Because the trees for migration rates between 0 and 1 are very similar, we only display five values of extinction (μ=0,0.15,1,5,1000) . The branches are colored by the location of species. Sympatric speciation and allopatric speciation are also distinguishable by the color of the nodes and the daughter species.

We simulated trees starting with two ancestral species, one in each region. We used the Gillespie algorithm (Gillespie 1976) to calculate the waiting time between two evolutionary events; this time is exponentially distributed with the sum of all rates as parameter. The probability of each event occurring is proportional to its rate relative to the sum of rates. A speciation event produces a new species, whereas an extinction event eliminates one existing species. Species dispersal and contraction do not change the number of species but alter the character of species, switching between endemic and widespread. The simulation is performed for a given amount of time (the crown age) and conditional on survival of the crown lineages (i.e., the simulation is restarted if one or both become extinct to guarantee that both ancestors have descendants at present) after which the phylogenetic tree of the extant species is constructed from the history of events. Here we show a series of trees (see Figs. 1 and S1 and S2 for trees under various scenarios to be discussed next) to demonstrate how trees are shaped under different parameter combinations.

We simulated the phylogenies under a variety of parameter values. To explore how the ecological limit to diversity affects the detection of the DD signal, we designed three spatial scenarios differing in ecological limits: two scenarios with identical limits on each location (Scenario 1: $K^{\prime }=20$, Scenario 2: $K^{\prime }=40$), and one scenario with different ecological limits (Scenario 3: $K_{1}^{\prime }=20,K_{2}^{\prime }=40$). For comparison with the nonspatial model, we additionally simulated two nonspatial scenarios differing in ecological limit (Scenario 4: $K^{\prime }=20$ and Scenario 5: $K^{\prime }=40$). We assumed a crown age of 15 time units, which can be interpreted as 15 million years. We fixed the values for the intrinsic speciation rates:

$$\lambda_{1,0}=\lambda_{2,0}=0.8,\;\lambda_{12,0}=0.2.$$

We looked at the same set of extinction rates as in (Etienne et al. 2011, 2016): 0,0.1,0.2,0.4. Finally, we studied the behavior of the model and the inference under a gradient of intrinsic dispersal rates: $M_{0}=0,0.05,0.1,0.15,0.3,0.5,1,5,1000$. The case $M_{0}=0$ corresponds to a birth–death process occurring on two independent locations. As *M*_0_ increases, the model tends toward the nonspatial model (with one important difference, see “Results”) and species at the tips become increasingly widespread species. In all, we simulated 36 parameter sets for each scenario. For each parameter set, we generated 100 phylogenetic trees.

### INFERENCE

We applied a bootstrap likelihood ratio test (Gudicha et al. 2016; Etienne et al. 2016; Tekle et al. 2016) to the simulated data to determine the power of the nonspatial model to detect diversity‐dependence in the spatial model. The χ^2^ likelihood ratio test cannot be used due to the mismatch between type I error rate and the significance level used as reported in Etienne et al. (2016). The bootstrap likelihood ratio test (Etienne et al. 2016) proceeds as follows:

(1) Collect an empirical dataset of phylogenetic branching times. One can also simulate data under another model for a specific parameter set (which was the case for our study in which we simulated under the spatial model).
(2) Estimate from these data the maximum‐likelihood (ML) parameters under the CR model and the DD model (the nonspatial model). Then calculate the likelihood ratio that is denoted by $LR_{0}$.
(3) Generate a bootstrap sample by simulating $X_{CR}$ datasets under the CR model using the parameter estimates obtained for the CR model in step 2.
(4) For each of these $X_{CR}$ simulated CR datasets, estimate the parameters under the CR model as well as the DD model and compute the likelihood ratio ($LR_{i}$ for dataset *i*).
(5) Compare the observed $LR_{0}$ with the distribution of $LR_{i}$‐values $(i=1..X_{CR}$) from the bootstrap simulations. Count the number of simulations with LR larger than $LR_{0}$ and denote the number by $R_{CR}$. The *p‐*value of the test is defined as $\left(R_{CR}+1\right)/\left(X_{CR}+1\right)$.
(6) A significance level α (e.g., 0.05) is set to accept or reject the CR model by comparison with the *P‐*value. Record the LR associated with this α, $LR_{\alpha }$.
(7) To assess the power of the test, simulate $X_{DD}$ times under the DD model with the ML parameters estimated under the DD model in step 2.
(8) For these $X_{DD}$ datasets simulated in step 7, estimate parameters under both CR and DD model and compute the LR for each dataset.
(9) The larger the number of the likelihood ratios exceeding $LR_{\alpha }$, the clearer is the signal of diversity‐dependence. Denote the number of the $X_{DD}$ simulations in which the LR is larger than $LR_{\alpha }$ by $R_{DD}$. Define the power of the test by $R_{DD}/\left(X_{DD}+1\right)$.

We performed this method for all the parameter sets. We thus have 36 parameter sets of 100 simulations each with 2000 bootstrap samples, totaling 7.2 million simulations and parameter estimations for each scenario. Given that each parameter estimation takes a few minutes, the total computation time for 5 scenarios was 50–100 million minutes, roughly, 100–200 years on a single computer. Hence, we performed these calculations on a high‐performance computing cluster, but even then computational time was substantial. We therefore provide all simulations and data as supplementary material.

## Results

### MODEL BEHAVIOR

**Figure 2:**
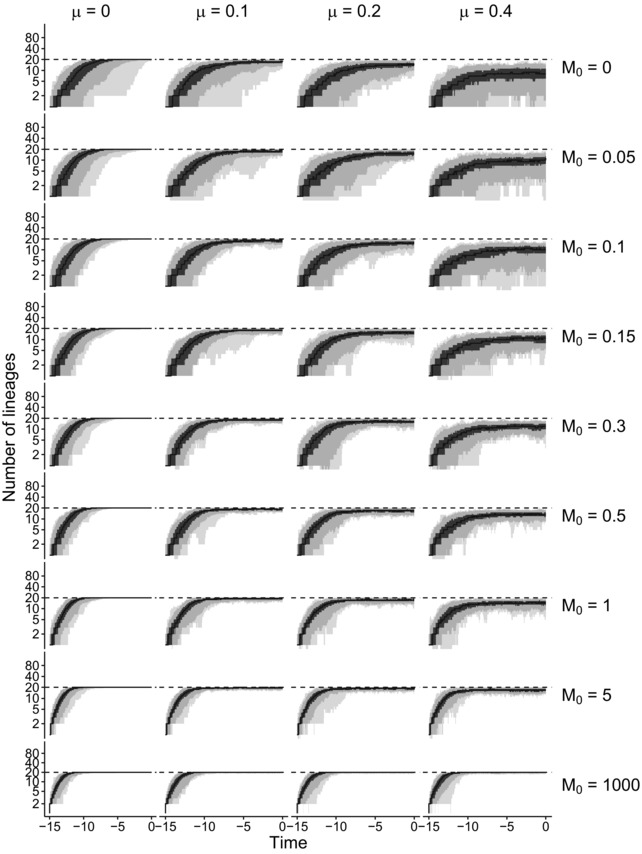
Species‐through‐time (STT) plots that include extinct species for one location across all parameter settings of Scenario 1. Lower extinction accelerates species accumulation. Species dispersal increases the number of species at equilibrium. The dashed line at value 20 shows the input value of $K^{\prime }$. The black line denotes the median STT plot, the gray shading represents the quantiles (minimum, 2.5th percentile, 25th percentile, 75th percentile, 97.5th percentiles, maximum).

To study how the model behaves under different dispersal and extinction rates, we plotted the species‐through‐time (STT) plots that include both extant and extinct species under different $K^{\prime }$ settings (see Fig. 2 for Scenario 1 and Figs. S7–S9 for other scenarios). The STT plots show how the total number of species changes due to macroevolutionary events. The STT plots that we show here are for a single location because in our model the diversity‐dependence is defined as local dynamics. We also plotted the nonspatial STT plots tracking the total number of species in the system, that is, for both locations together as supplementary results (see Figs. S10–S12). As expected, from the local STT plots we observed a positive correlation between species dispersal and species richness and a negative correlation between extinction and species richness. However, in the nonspatial STT plots dispersal seems to have a complex influence on the global species richness. Although the effect of dispersal is small, it gets larger with increasing extinction rate. We will discuss it later in the section of parameter estimation. To test the model behavior under high species dispersal rate, we additionally explored an extreme case in which dispersal rate is extremely large ($M_{0}=1000$). In this case, all parameter settings varying only in extinction rates lead a similar increasing pattern in species richness and the diversity in both locations reach the ecological limit rapidly (e.g., $K^{\prime }=20$ for Scenario 1, see Figs. 2 and S7–S9 for other scenarios). This phenomenon is similar to a pure birth process due to the extremely high dispersal rate. The biological explanation is that once an endemic species is produced, it spreads out to the other location immediately, which makes it almost impossible to go globally extinct. Therefore, the system is filled with widespread species and a few endemic species at the equilibrium level, which is identical to the ecological limit.

**Figure 3:**
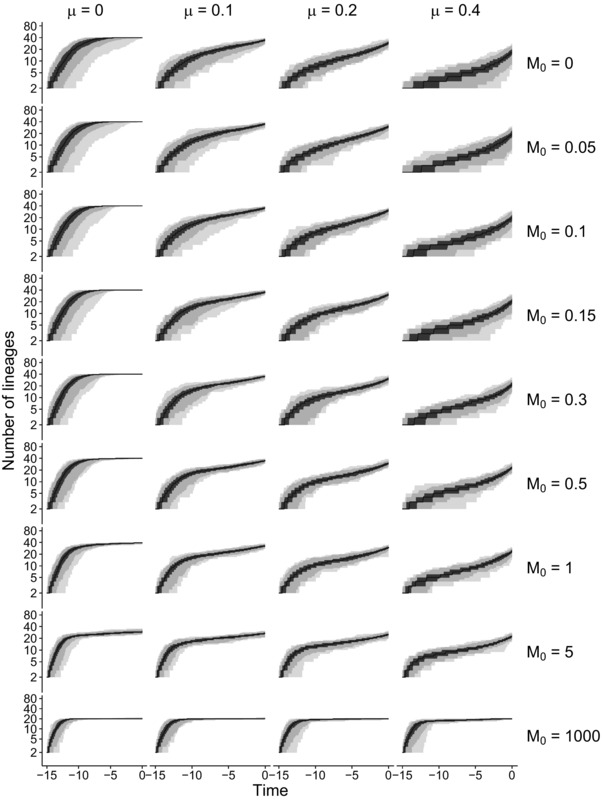
Lineages‐through‐time (LTT) plots that only include extant species and their ancestors across 100 simulations for each explored parameter combination of Scenario 1. The black line denotes the median STT plot, the gray shading represents the quantiles (minimum, 2.5th percentile, 25th percentile, 75th percentile, 97.5th percentiles, maximum).

Furthermore, we studied LTT plots for extant species for both locations together, which allows comparison with LTT plots from the nonspatial model. We observed a pattern of an early burst and the pull of the present (Nee et al. 1994; Kubo and Iwasa 1995; Fig. 3 for Scenario 1 and Figs. S13–S14 for other scenarios), except for the highest extinction rate (μ=0.4) and lowest dispersal rate ($M_{0}=0$), for which the shape of the LTT plot approaches a straight line.

### DETECTING DIVERSITY‐DEPENDENCE

Diversity‐dependence can be detected with high power except when extinction is high (larger than 0.4) and species dispersal is low (smaller than 1) at the significance level α=0.05 (see Fig. 5 for Scenario 1 and Figs. S5 and S6 for other scenarios). This suggests that extinction tends to erase the signature of diversity‐dependence, while species dispersal strengthens the signal. When relating this to the STT and LTT plots, we observe that weak signals of diversity‐dependence are accompanied with a low rate of species accumulation. In contrast, strong evidence for diversity‐dependence often occurs for low extinction and high dispersal. Both these situations lead to intense species interactions. We also observe substantial early bursts for LTT plots whenever diversity‐dependence is detected.

**Figure 4:**
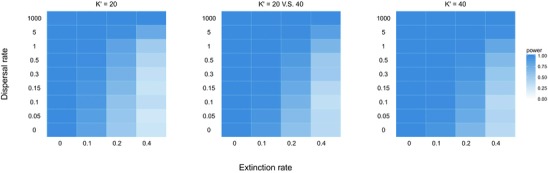
Power of the diversity‐dependence detection for three spatial scenarios. The dark blue color denotes high power of diversity‐dependence, light blue denotes low power.

To explore whether the DD signal would be stronger in the scenario that has a higher ecological limit to diversity, we studied the power of the test for different scenarios with different ecological limits. Figure 4 shows power to detect diversity‐dependence under different parameter combinations of three spatial scenarios. We observe that systems with a higher ecological limit to diversity show a broader range of high detection power in parameter space. In particular, the scenario with distinct limits ($K_{1}^{\prime }=20,K_{2}^{\prime }=40$) on two locations shows an intermediate strength of diversity‐dependence between two scenarios with identical limits, stronger than Scenario 1 ($K^{\prime }=20$) and weaker than Scenario 2 ($K^{\prime }=40$).

**Figure 5:**
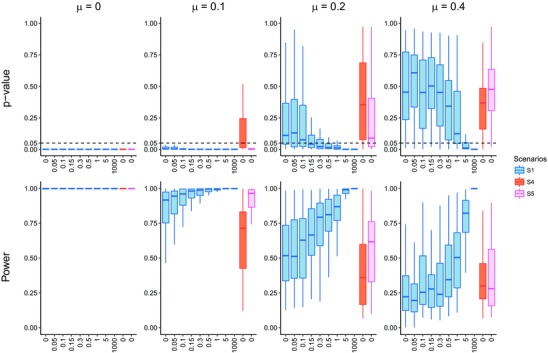
*P*‐values and powers of the test of spatial Scenario 1 and nonspatial Scenarios 4 and 5: as the dispersal rate increases, the *P*‐value declines approaching 0 while the power of the test rises up to 1. The signal of diversity‐dependence tends to be detected with high dispersal and low extinction. Especially, in the case of the pure birth process, all the scenarios show such a strong signal that the distribution bars of *P*‐values and powers are compressed to thick black lines. When extinction rate is 0.4, diversity‐dependence is not detected statistically until dispersal rate reaches 1. In the box plots, thick solid lines, boxes and whiskers denote the percentiles of 50, 75, and 95%, respectively.

We next explored whether the partition of the community into two locations would weaken the strength of the DD signal. The nonspatial Scenarios 4 and 5 have the same value of ecological limits as the spatial Scenarios 1 and 2, respectively, but constrain the species diversification to only one single location. The spatial structure indeed affects the diversity‐dependence detection but in a complex manner (see Figs. 5 and S5 and S6 for other spatial scenarios). When the locations are more isolated, that is, they have little species interaction between them, the nonspatial scenarios show stronger diversity‐dependence than the spatial scenarios. When dispersal rate increases, this pattern is reversed, because species dispersal reduces extinction thus leading to a high rate of species accumulation.

### PARAMETER ESTIMATE ACCURACY AND PRECISION

**Figure 6:**
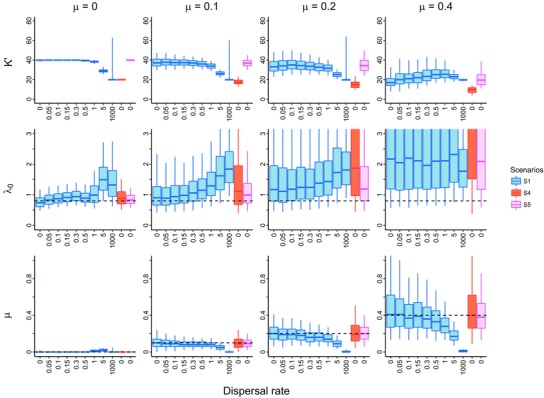
Maximum‐likelihood estimates for the ecological limit parameter $K^{\prime }$, speciation rate and extinction rate for all the parameter settings of spatial Scenario 1 versus nonspatial Scenarios 4 and 5. The dashed lines indicate the values used in the simulations. In the box plots, thick solid lines, boxes, and whiskers denote the 50, 75, and 95% percentiles, respectively.

The performance of parameter estimation depends strongly on the extinction and dispersal rates. Accurate parameter estimations are obtained for low extinction and dispersal. The median estimates for the ecological limit are around the sum of the local limits (Figs. 6 and S3 and S4 for other scenarios) when both extinction and dispersal rates are low. But bias in parameter estimates increases for larger dispersal and extinction rates. This is due to the fact that both dispersal and extinction strongly control the species richness of the system. Extinction has a negative effect on diversity so we find that our estimate of the ecological limit decreases with increasing extinction. The influence of dispersal on species richness is more complex. On the one hand, dispersal promotes the conversion of endemic species to widespread species thereby decreasing species richness. On the other hand, dispersal reduces extinction and thereby increases species richness. We observe this phenomenon in our simulation study, especially for high extinction. In all scenarios, the estimates of the ecological limit increase at first but then drop with the dispersal rate increases. This also explains the pattern that the equilibrium of species richness in the nonspatial STT plots first increases and then declines with increasing dispersal rate.

Speciation and extinction estimates are robust when both extinction and dispersal rates are low. However, when species dispersal increases the speciation estimates are biased upward while extinction is biased downward. Interestingly, the speciation estimates are biased up to a value equal to the sum of the local speciation rates of the two locations. The extinction estimates are biased down to zero, which agrees with the explanation that dispersal reduces extinction.

We also tested the influence of diversity on parameter estimation. Through comparing among scenarios with varying ecological limits, we found that higher species diversity leads to less variation in estimates. This is also true for simulations with the nonspatial model.

## Discussion

DD diversification has long been recognized as a potential explanation for slowdowns in species accumulation (Weir 2006; Phillimore and Price 2008; Rabosky and Lovette 2008a; Rundell and Price 2009; Rabosky 2013). Methods to estimate model parameters from phylogenetic trees exist (Etienne et al. 2011; Etienne and Haegeman 2012) but have not yet fully addressed the question: if diversity‐dependence is operating, can it be reliably detected? Etienne et al. (2016) looked at simulations with the nonspatial DD model and studied when the presence or absence of diversity‐dependence can be detected using the likelihood derived for this nonspatial model. In this article, we take a further step to explore if this nonspatial likelihood approach is still applicable when data are generated by a spatial model in which diversity‐dependence occurs at a local scale. We developed a spatial DD diversification model that incorporates species interactions between two locations. Our spatial DD diversification model advances existing phylogenetic tools by integrating spatial dynamics and lineage diversification processes that depend on species richness. While models combining biogeography and macroevolutionary diversification are already available (Nepokroeff et al. 2003; Sanmartín et al. 2008; Goldberg et al. 2011), our model is the first to incorporate diversity‐dependence.

We demonstrated that the method based on the nonspatial diversity‐dependence model can detect local diversity‐dependence simulated under our spatial model, except when dispersal is rare or extinction is high. Extinction weakens and dispersal strengthens the signal of diversity‐dependence. Variability between simulations decreases somewhat with lower extinction, but more so with higher dispersal rate. Hence, stochasticity due to extinctions is less prominent than stochasticity due to asynchrony between locations. When extinction is high, diversity‐dependence detection is difficult, but this is also true when the data are generated by the nonspatial model itself (Etienne et al. 2016), so this is not caused by the difference between generating and inference model per se. The STT plots suggest that this is because diversity is relatively low during a large part of the macroevolutionary history, and hence diversity‐dependence was nearly absent. Parameter estimates were biased and more so for higher extinction rates. Again, this bias caused by extinction was also found when generating and inference model were both nonspatial (Etienne et al. 2016).

Our results reveal the influence of geographic structure and species diversity on the diversity‐dependence detection. Comparing statistical power among the three spatial scenarios, we found that the strength of the diversity‐dependence detection depends mostly on the species diversity of the community regardless of the specific limits of the locations. This higher power is simply because with larger $K^{\prime }$ trees are larger and thus contain more information. However, it does not mean that diversity‐dependence itself is stronger. Diversity‐dependence only really affects diversification when the diversity is close to equilibrium. If the ecological limit is too large to allow equilibrium to be reached within the given time (the crown age), diversity‐dependence will have little effect on diversification. Hence, we expect that diversity‐dependence detection becomes more difficult when we increase $K^{\prime }$ to such values that equilibrium is still far away with the given time. Our comparison between spatial scenarios and nonspatial scenarios demonstrated the negative effect of spatial partitioning on the power of diversity‐dependence detection but only when dispersal rate is low. This is mainly because we do not allow global extinction for widespread species, and thus increasing dispersal rate reduces extinction thereby promoting species richness. If we allowed for global extinction, we would expect the power of detecting diversity‐dependence in spatial scenarios with large dispersal rate to approach the power in nonspatial scenarios. But new issues will then arise: how do we define global extinction, and how can we distinguish between global extinction and local extinction? This will depend on the type of extinction. For example, extinction caused by natural disasters may be operating mostly on a local scale and are therefore independent between regions. By contrast, extinction caused by an infectious disease is likely correlated with dispersal, and hence global and local extinction are linked. Because such complex mechanisms are not easy to incorporate into the relatively simple model that we consider, we assumed a model with uncorrelated and constant extinction (but see Ezard et al. 2011; Sanmartín and Meseguer 2016).

Our two‐location model is the simplest case of a multiple‐location model. A more general model for any number of locations is required to explore if local diversity‐dependence can reliably be detected. To perform the same kind of analysis with such a model as we did here for two locations, we face two main challenges. First, our simulations use the Gillespie algorithm to determine the waiting time between two evolutionary events; because more locations imply more events, the sum of all event rates will become extremely large and hence the waiting time will become extremely short resulting in simulations taking a very long time. Second, the spatial arrangement of multiple locations affects dispersal patterns and thereby the results of our model. Hence, we would have to explore many spatial arrangements of the locations. Based on our results for two locations, we expect that diversity‐dependence will be detected well when dispersal is not too low and extinction is not too high, where the power of the detection method will depend subtly on the spatial arrangement.

Even with many locations, the model remains only a coarse approximation to reality. We do not model species interactions mechanistically, but simply define a phenomenological carrying capacity, but, importantly, on a local scale. The literature on competition models is huge, so the question is where one would start to explore the robustness of our approach to varying the underlying competition mechanisms. We suggest to move toward mechanistic models in steps that are small in terms of model structure, but large in their conceptual difference. For example, one could incorporate an influence of phylogenetic relatedness on interaction strength. Phylogenetic structure emerges from our model itself, so this relatively small change in model structure implies an interesting feedback mechanism that is a major conceptual change. Another example would be to include trait evolution and trait‐dependent competition. These mechanisms, however, still imply a local carrying capacity, and we therefore expect that our results will hold up in more complex, but more realistic models.

Our results provide context for the empirical scientists who want to apply the nonspatial inference tool to her or his phylogeny. We have shown that even if the species in the phylogeny are spatially distributed, the nonspatial tool is able to tell whether ecology (diversity) is limiting diversification. Only when a high extinction combined with low dispersal is expected, then some caution is needed. Furthermore, the parameters inferred using the nonspatial tool bear some relationship to the real processes, but should not be interpreted too literally.

Associate Editor: I. Sanmartín

Handling Editor: P. Tiffin

## Supporting information


**Fig. S1**. A list of phylogenetic trees of Scenario 2.
**Fig. S2**. A list of phylogenetic trees of Scenario 3.
**Fig. S3**. Parameter estimations for Scenario 2 versus Scenarios 4 and 5.
**Fig. S4**. Parameter estimations for Scenario 3 versus Scenarios 4 and 5.
**Fig. S5**. *P*‐values and powers of the test of spatial Scenario 2 versus non‐spatial Scenarios 4 and 5.
**Fig. S6**. *P*‐values and powers of the test of spatial Scenario 3 versus non‐spatial Scenarios 4 and 5.
**Fig. S7**. Local species‐through‐time (STT) plots of Scenario 2 on location 1.
**Fig. S8**. Local species‐through‐time (STT) plots of Scenario 3 on location 1.
**Fig. S9**. Local species‐through‐time (STT) plots of Scenario 3 on location 2.
**Fig. S10**. Nonspatial species‐through‐time (STT) plots of Scenario 1.
**Fig. S11**. Nonspatial species‐through‐time (STT) plots of Scenario 2.
**Fig. S12**. Nonspatial species‐through‐time (STT) plots of Scenario 3.
**Fig. S13**. Lineages‐through‐time (LTT) plots of Scenario 2.
**Fig. S14**. Lineages‐through‐time (LTT) plots of Scenario 3.
