# Supplementary material for: Detecting local diversity‐dependence in diversification
Source: Evolution. 2018 Apr 24;72(6):1294–305. doi: 10.1111/evo.13482 (PMC6055638; doi:10.1111/evo.13482)
Supplement: Supplementary file 1 — Fig. S1. A list of phylogenetic trees of Scenario 2. Fig. S2. A list of phylogenetic trees of Scenario 3. Fig. S3. Parameter estimations for Scenario 2 versus Scenarios 4 and 5. Fig. S4. Parameter estimations for Scenario 3 versus Scenarios 4 and 5. Fig. S5. P‐values and powers of the test of spatial Scenario 2 versus non‐spatial Scenarios 4 and 5. Fig. S6. P‐values and powers of the test of spatial Scenario 3 versus non‐spatial Scenarios 4 and 5. Fig. S7. Local species‐through‐time (STT) plots of Scenario 2 on location 1. Fig. S8. Local species‐through‐time (STT) plots of Scenario 3 on location 1. Fig. S9. Local species‐through‐time (STT) plots of Scenario 3 on location 2. Fig. S10. Nonspatial species‐through‐time (STT) plots of Scenario 1. Fig. S11. Nonspatial species‐through‐time (STT) plots of Scenario 2. Fig. S12. Nonspatial species‐through‐time (STT) plots of Scenario 3. Fig. S13. Lineages‐through‐time (LTT) plots of Scenario 2. Fig. S14. Lineages‐through‐time (LTT) plots of Scenario 3. [file EVO-72-1294-s001.zip › evo13482-sup-0001-SuppMat.pdf]

Supporting Information

Supplementary results can be found in the supplementary material for this article:

We simulated the phylogenies under a variety of parameter values. To explore how the ecological limit to diversity affects the detection of the diversity-dependent signal, we designed three spatial scenarios differing in ecological limits: two scenarios with identical limits on each location(Scenario 1: $K' = 20$, Scenario 2: $K' = 40$), and one scenario with different ecological limits (Scenario 3:$K'_1 = 20, K'_2 = 40$). For comparison with the non-spatial model, we additionally simulated two non-spatial scenarios differing in ecological limit Scenario 4: $K' = 20$ and Scenario 5: $K' = 40$). We assumed a crown age of 15 time units, which can be interpreted as 15 million years. We fixed the values for the intrinsic speciation rates:

$$\lambda_{1,0} = \lambda_{2,0} = 0.8,\ \lambda_{12,0} = 0.2.$$

We looked at the same set of extinction rates as in (Etienne et al. 2011, 2016): $0, 0.1, 0.2, 0.4$. Finally, we studied the behavior of the model and the inference under a gradient of intrinsic dispersal rates: $M_0 = 0, 0.05, 0.1, 0.15, 0.3, 0.5, 1, 5, 1000$. The case $M_0 = 0$ corresponds to a birth-death process occurring on two independent locations. As M_0 increases, the model tends towards the non-spatial model (with one important difference, see Results) and species at the tips become increasingly widespread species. In all, we simulated 36 parameter sets for each scenario. For each parameter set, we generated 100 phylogenetic trees.

Fig.S1. A list of phylogenetic trees of Scenario 2.

Fig.S2. A list of phylogenetic trees of Scenario 3.

Fig.S3. Parameter estimations for Scenario 2 vs. Scenario 4 and 5.

Fig.S4. Parameter estimations for Scenario 3 vs. Scenario 4 and 5.

Fig.S5. P-values and powers of the test of spatial scenario 2 vs. non-spatial scenario 4 and 5.

Fig.S6. P-values and powers of the test of spatial scenario 3 vs. non-spatial scenario 4 and 5.

Fig.S7. Local species-through-time (STT) plots of Scenario 2 on location 1.

Fig.S8. Local species-through-time (STT) plots of Scenario 3 on location 1.

Fig.S9. Local species-through-time (STT) plots of Scenario 3 on location 2.

Fig.S10. Non-spatial species-through-time (STT) plots of Scenario 1.

Fig.S11. Non-spatial species-through-time (STT) plots of Scenario 2.

Fig.S12. Non-spatial species-through-time (STT) plots of Scenario 3.

Fig.S13. Lineages-through-time (LTT) plots of Scenario 2.

Fig.S14. Lineages-through-time (LTT) plots of Scenario 3.
