# Supplementary figures and images for: Detecting local diversity‐dependence in diversification
Source: Evolution. 2018 Apr 24;72(6):1294–305. doi: 10.1111/evo.13482 (PMC6055638; doi:10.1111/evo.13482)

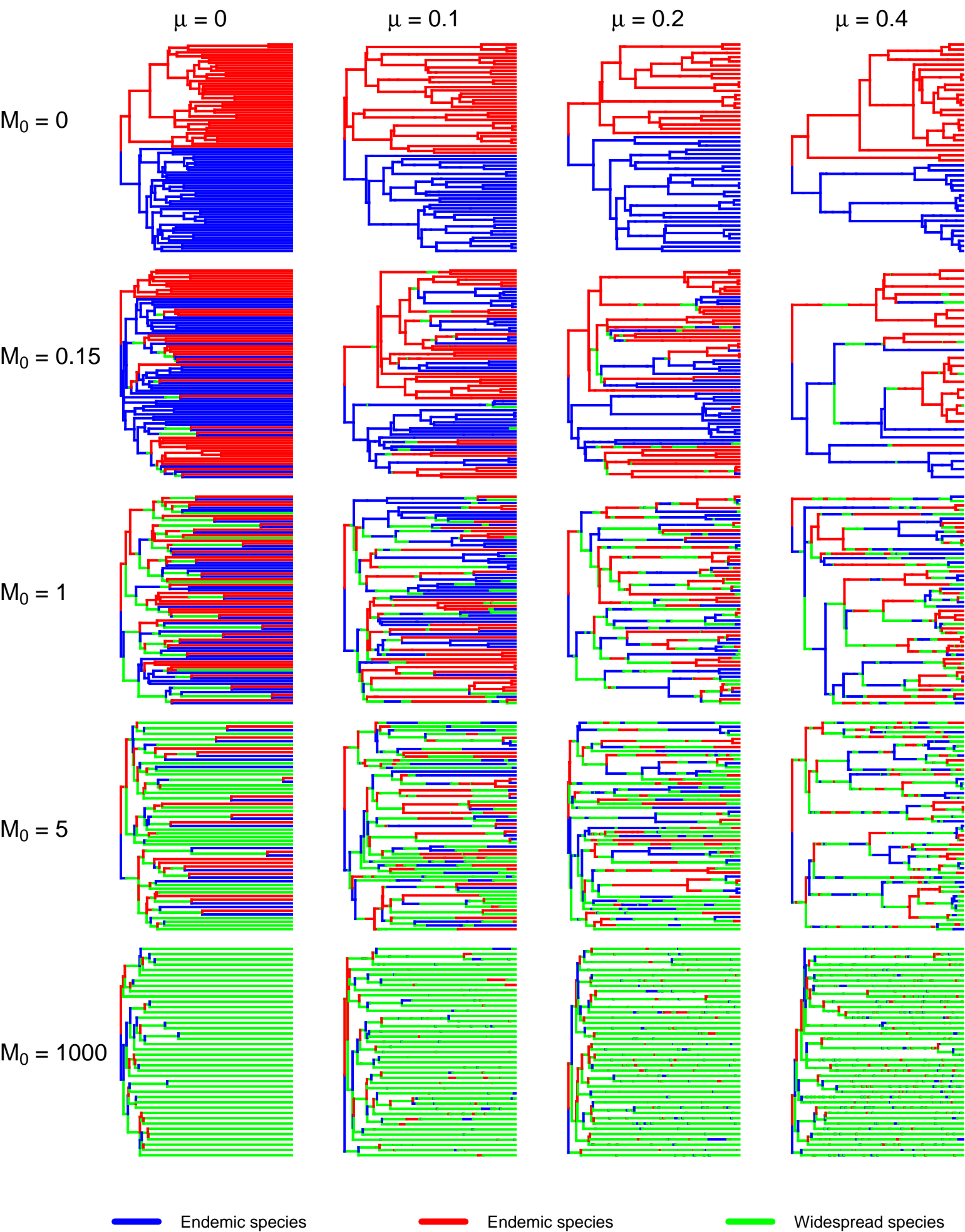

Supplement: Supplementary file 1 — Fig. S1. A list of phylogenetic trees of Scenario 2. Fig. S2. A list of phylogenetic trees of Scenario 3. Fig. S3. Parameter estimations for Scenario 2 versus Scenarios 4 and 5. Fig. S4. Parameter estimations for Scenario 3 versus Scenarios 4 and 5. Fig. S5. P‐values and powers of the test of spatial Scenario 2 versus non‐spatial Scenarios 4 and 5. Fig. S6. P‐values and powers of the test of spatial Scenario 3 versus non‐spatial Scenarios 4 and 5. Fig. S7. Local species‐through‐time (STT) plots of Scenario 2 on location 1. Fig. S8. Local species‐through‐time (STT) plots of Scenario 3 on location 1. Fig. S9. Local species‐through‐time (STT) plots of Scenario 3 on location 2. Fig. S10. Nonspatial species‐through‐time (STT) plots of Scenario 1. Fig. S11. Nonspatial species‐through‐time (STT) plots of Scenario 2. Fig. S12. Nonspatial species‐through‐time (STT) plots of Scenario 3. Fig. S13. Lineages‐through‐time (LTT) plots of Scenario 2. Fig. S14. Lineages‐through‐time (LTT) plots of Scenario 3. [file EVO-72-1294-s001.zip › evo13482-sup-0003-Trees_S2.pdf]

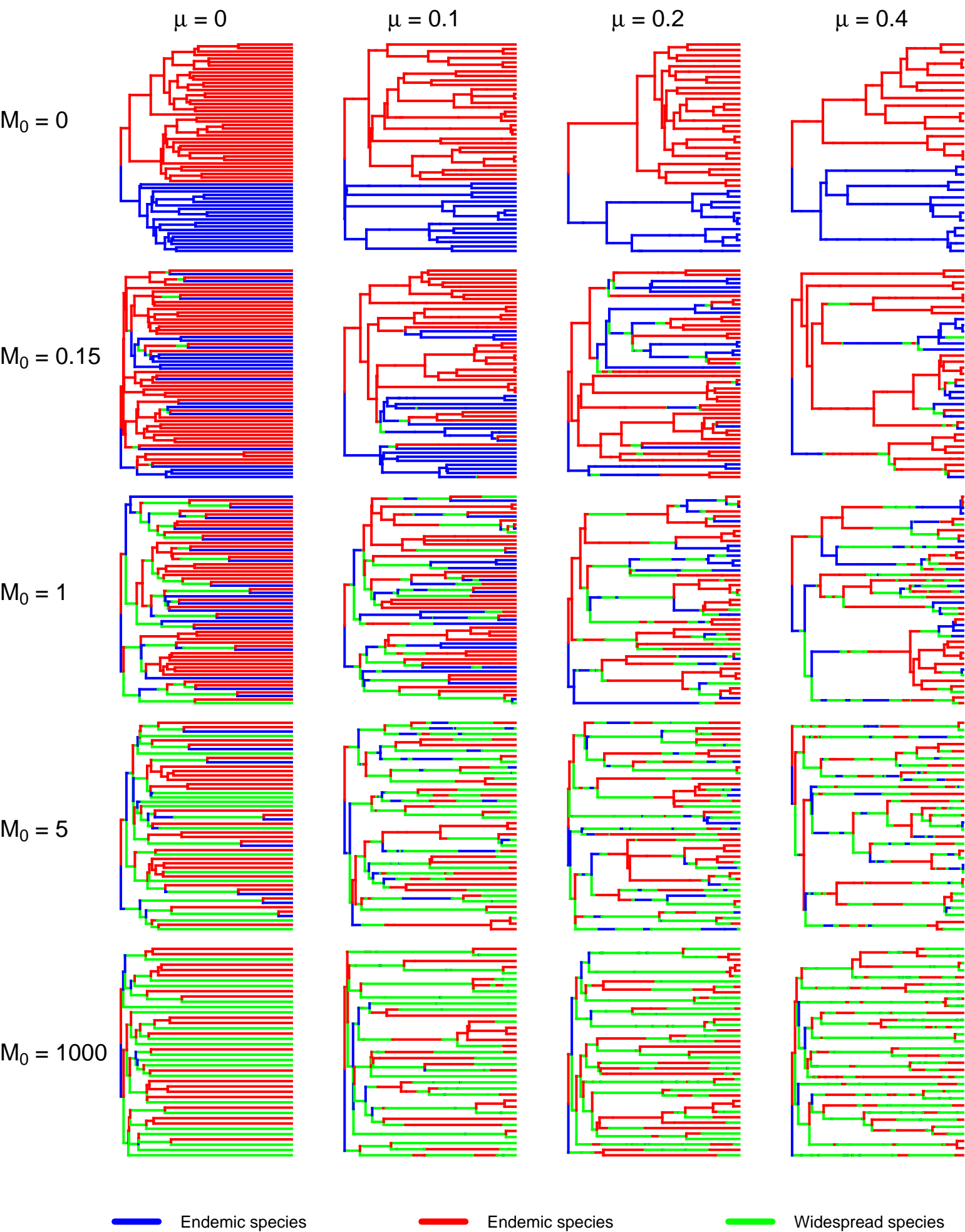

Supplement: Supplementary file 1 — Fig. S1. A list of phylogenetic trees of Scenario 2. Fig. S2. A list of phylogenetic trees of Scenario 3. Fig. S3. Parameter estimations for Scenario 2 versus Scenarios 4 and 5. Fig. S4. Parameter estimations for Scenario 3 versus Scenarios 4 and 5. Fig. S5. P‐values and powers of the test of spatial Scenario 2 versus non‐spatial Scenarios 4 and 5. Fig. S6. P‐values and powers of the test of spatial Scenario 3 versus non‐spatial Scenarios 4 and 5. Fig. S7. Local species‐through‐time (STT) plots of Scenario 2 on location 1. Fig. S8. Local species‐through‐time (STT) plots of Scenario 3 on location 1. Fig. S9. Local species‐through‐time (STT) plots of Scenario 3 on location 2. Fig. S10. Nonspatial species‐through‐time (STT) plots of Scenario 1. Fig. S11. Nonspatial species‐through‐time (STT) plots of Scenario 2. Fig. S12. Nonspatial species‐through‐time (STT) plots of Scenario 3. Fig. S13. Lineages‐through‐time (LTT) plots of Scenario 2. Fig. S14. Lineages‐through‐time (LTT) plots of Scenario 3. [file EVO-72-1294-s001.zip › evo13482-sup-0004-Trees_S3.pdf]

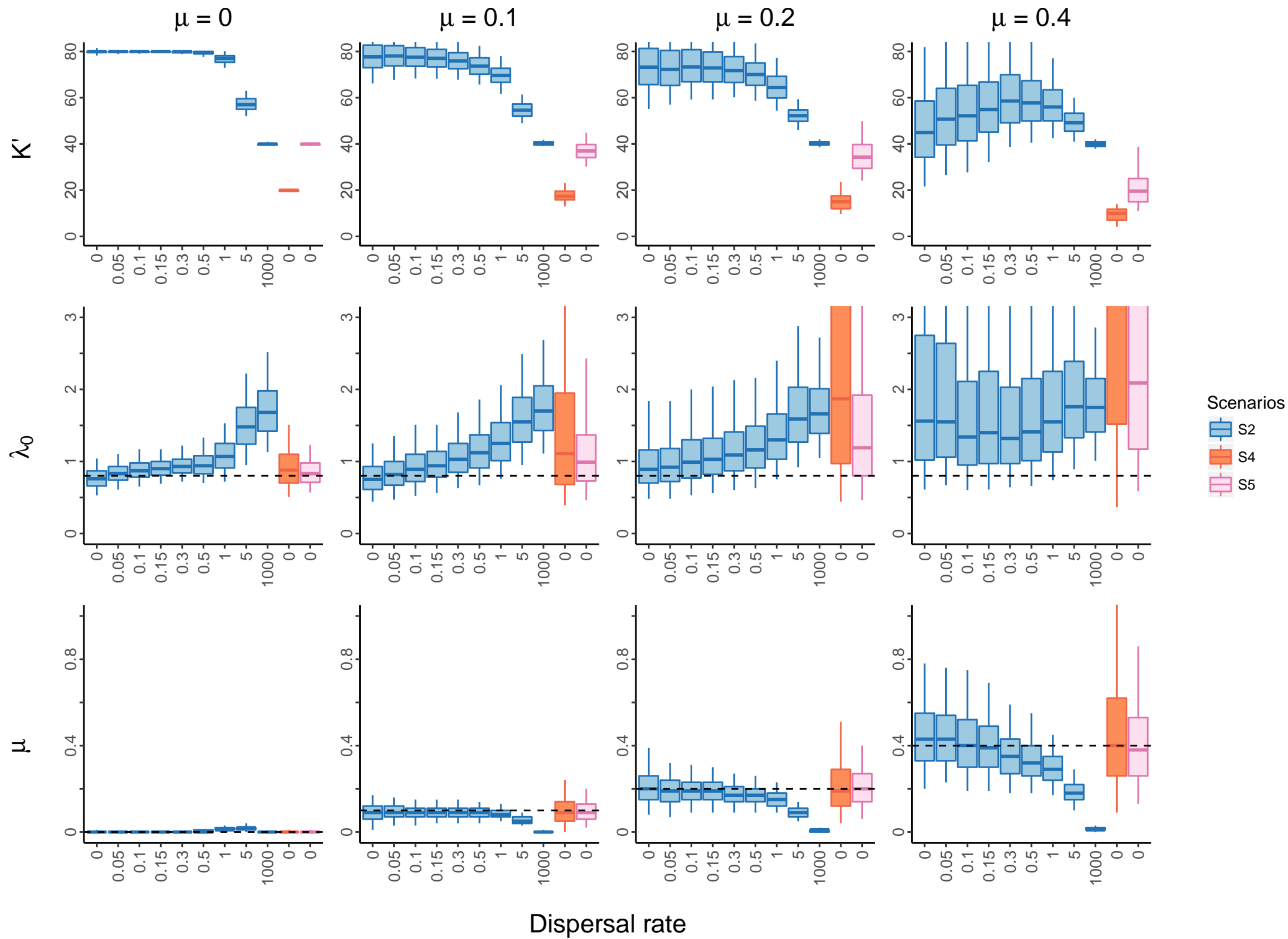

Supplement: Supplementary file 1 — Fig. S1. A list of phylogenetic trees of Scenario 2. Fig. S2. A list of phylogenetic trees of Scenario 3. Fig. S3. Parameter estimations for Scenario 2 versus Scenarios 4 and 5. Fig. S4. Parameter estimations for Scenario 3 versus Scenarios 4 and 5. Fig. S5. P‐values and powers of the test of spatial Scenario 2 versus non‐spatial Scenarios 4 and 5. Fig. S6. P‐values and powers of the test of spatial Scenario 3 versus non‐spatial Scenarios 4 and 5. Fig. S7. Local species‐through‐time (STT) plots of Scenario 2 on location 1. Fig. S8. Local species‐through‐time (STT) plots of Scenario 3 on location 1. Fig. S9. Local species‐through‐time (STT) plots of Scenario 3 on location 2. Fig. S10. Nonspatial species‐through‐time (STT) plots of Scenario 1. Fig. S11. Nonspatial species‐through‐time (STT) plots of Scenario 2. Fig. S12. Nonspatial species‐through‐time (STT) plots of Scenario 3. Fig. S13. Lineages‐through‐time (LTT) plots of Scenario 2. Fig. S14. Lineages‐through‐time (LTT) plots of Scenario 3. [file EVO-72-1294-s001.zip › evo13482-sup-0005-Est.S2VS.pdf]

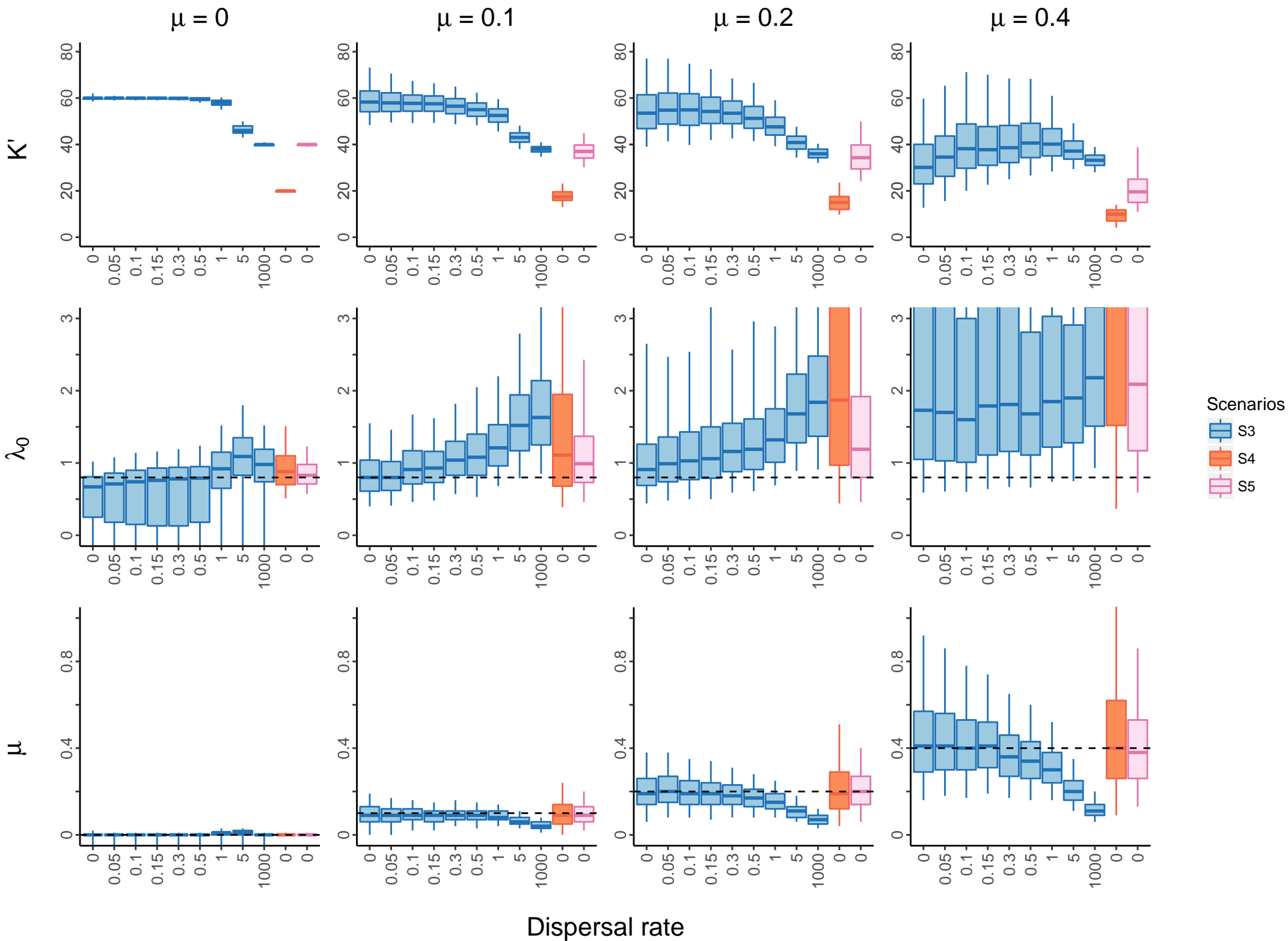

Supplement: Supplementary file 1 — Fig. S1. A list of phylogenetic trees of Scenario 2. Fig. S2. A list of phylogenetic trees of Scenario 3. Fig. S3. Parameter estimations for Scenario 2 versus Scenarios 4 and 5. Fig. S4. Parameter estimations for Scenario 3 versus Scenarios 4 and 5. Fig. S5. P‐values and powers of the test of spatial Scenario 2 versus non‐spatial Scenarios 4 and 5. Fig. S6. P‐values and powers of the test of spatial Scenario 3 versus non‐spatial Scenarios 4 and 5. Fig. S7. Local species‐through‐time (STT) plots of Scenario 2 on location 1. Fig. S8. Local species‐through‐time (STT) plots of Scenario 3 on location 1. Fig. S9. Local species‐through‐time (STT) plots of Scenario 3 on location 2. Fig. S10. Nonspatial species‐through‐time (STT) plots of Scenario 1. Fig. S11. Nonspatial species‐through‐time (STT) plots of Scenario 2. Fig. S12. Nonspatial species‐through‐time (STT) plots of Scenario 3. Fig. S13. Lineages‐through‐time (LTT) plots of Scenario 2. Fig. S14. Lineages‐through‐time (LTT) plots of Scenario 3. [file EVO-72-1294-s001.zip › evo13482-sup-0006-Est.S3VS.pdf]

$\mu = 0$  $\mu = 0.1$  $\mu = 0.2$  $\mu = 0.4$ 

p-value

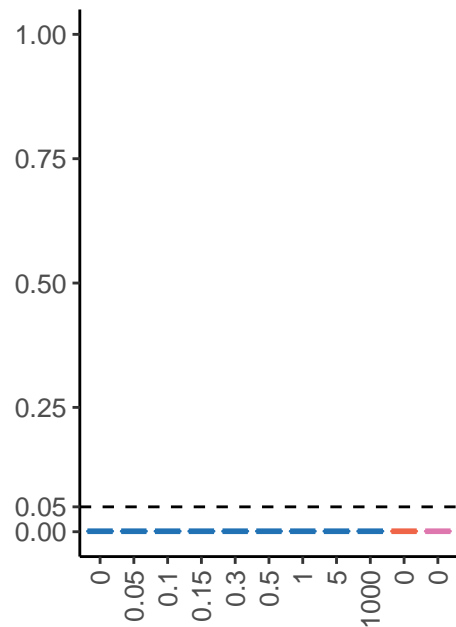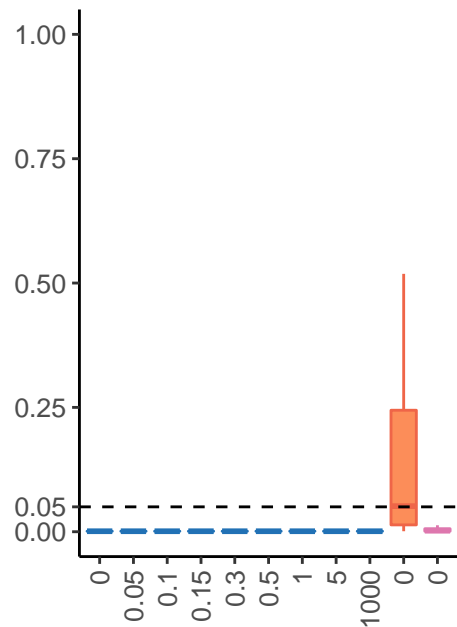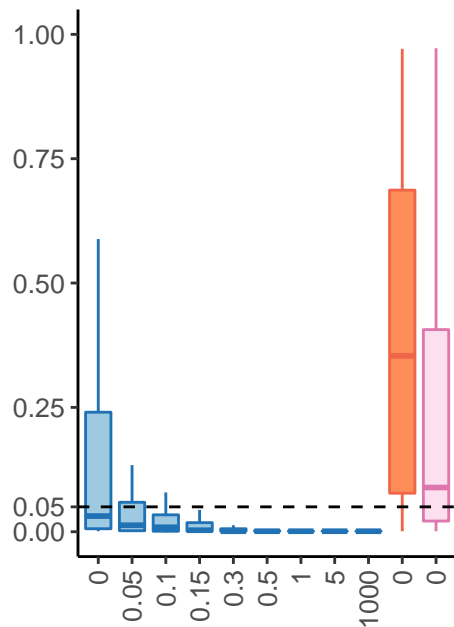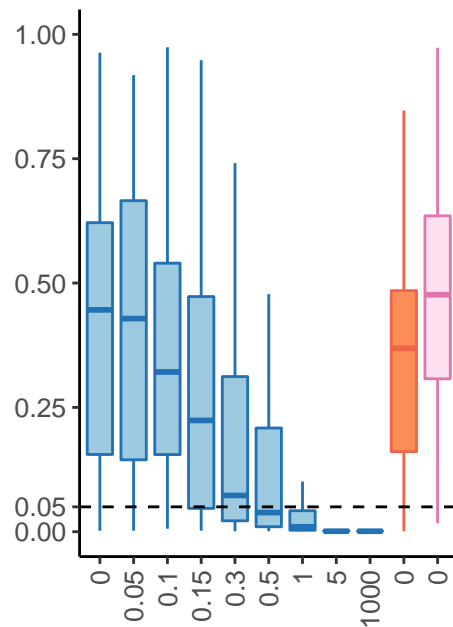

Power

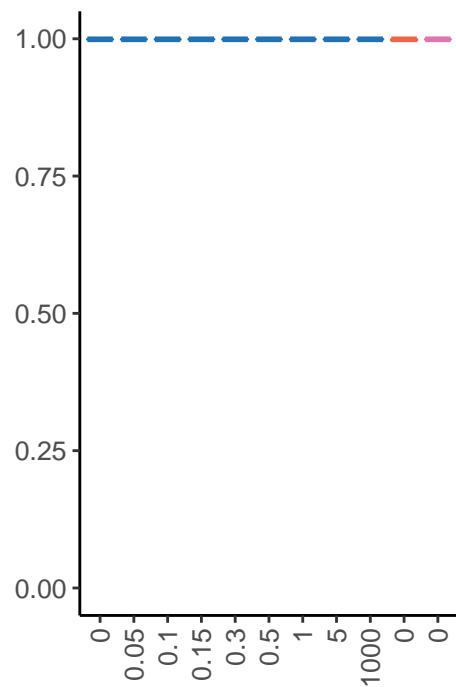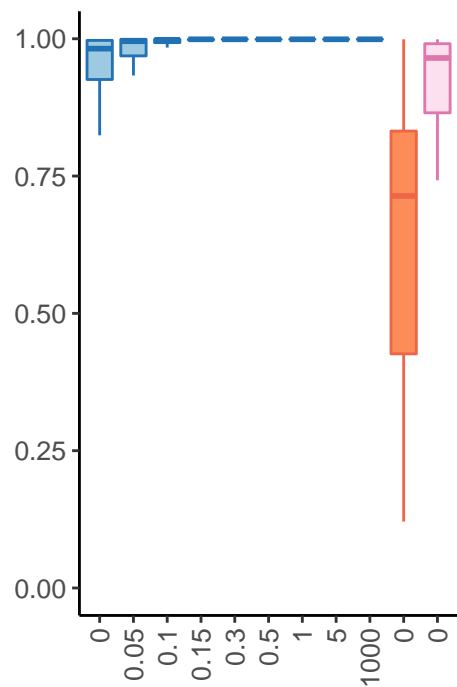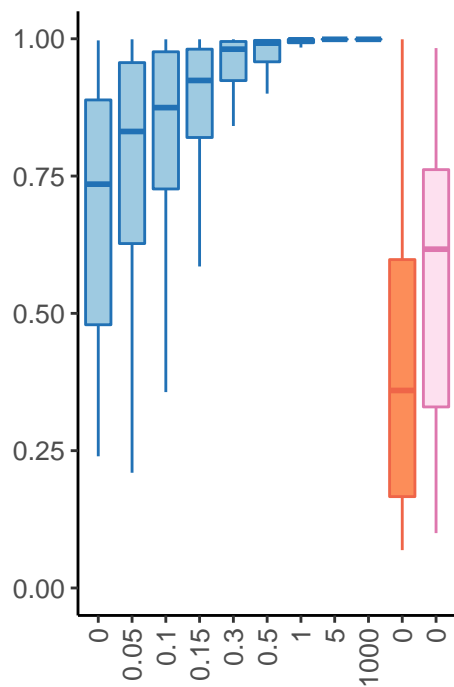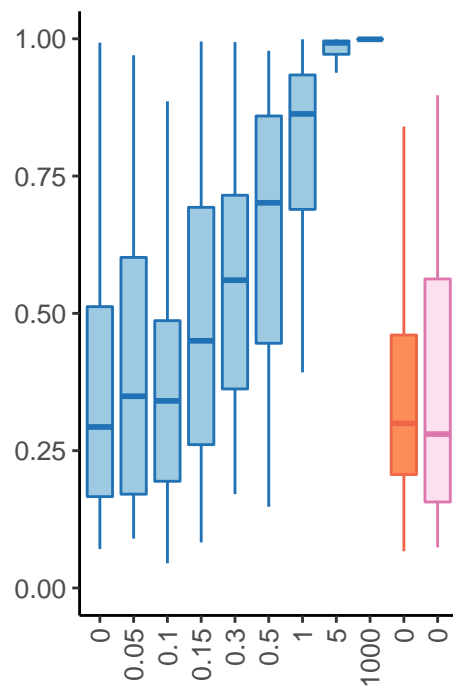

Scenarios

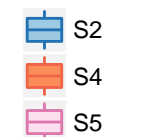

Dispersal rate

Supplement: Supplementary file 1 — Fig. S1. A list of phylogenetic trees of Scenario 2. Fig. S2. A list of phylogenetic trees of Scenario 3. Fig. S3. Parameter estimations for Scenario 2 versus Scenarios 4 and 5. Fig. S4. Parameter estimations for Scenario 3 versus Scenarios 4 and 5. Fig. S5. P‐values and powers of the test of spatial Scenario 2 versus non‐spatial Scenarios 4 and 5. Fig. S6. P‐values and powers of the test of spatial Scenario 3 versus non‐spatial Scenarios 4 and 5. Fig. S7. Local species‐through‐time (STT) plots of Scenario 2 on location 1. Fig. S8. Local species‐through‐time (STT) plots of Scenario 3 on location 1. Fig. S9. Local species‐through‐time (STT) plots of Scenario 3 on location 2. Fig. S10. Nonspatial species‐through‐time (STT) plots of Scenario 1. Fig. S11. Nonspatial species‐through‐time (STT) plots of Scenario 2. Fig. S12. Nonspatial species‐through‐time (STT) plots of Scenario 3. Fig. S13. Lineages‐through‐time (LTT) plots of Scenario 2. Fig. S14. Lineages‐through‐time (LTT) plots of Scenario 3. [file EVO-72-1294-s001.zip › evo13482-sup-0007-PP.S2VS.pdf]

$\mu = 0$  $\mu = 0.1$  $\mu = 0.2$  $\mu = 0.4$ 

p-value

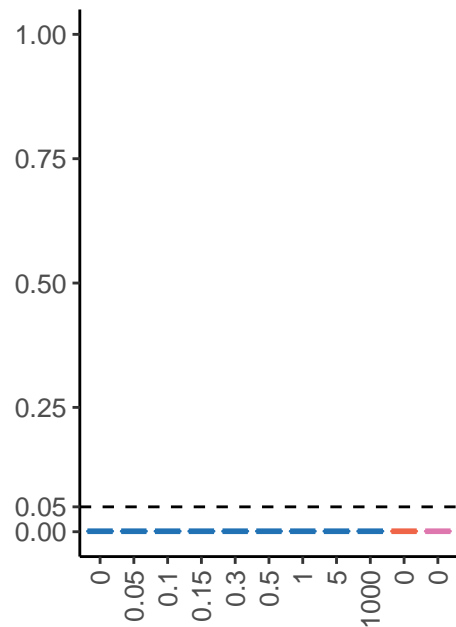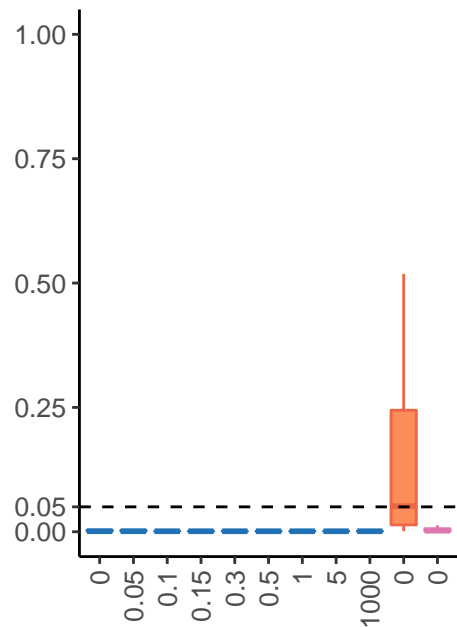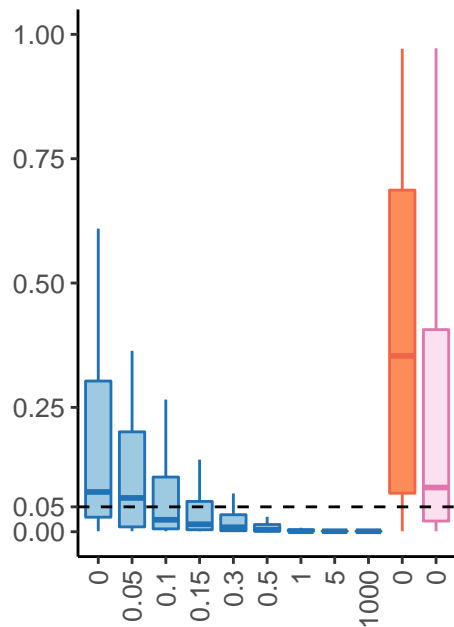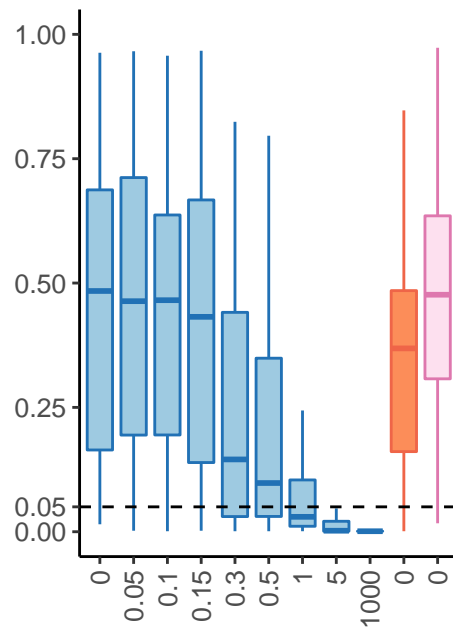

Power

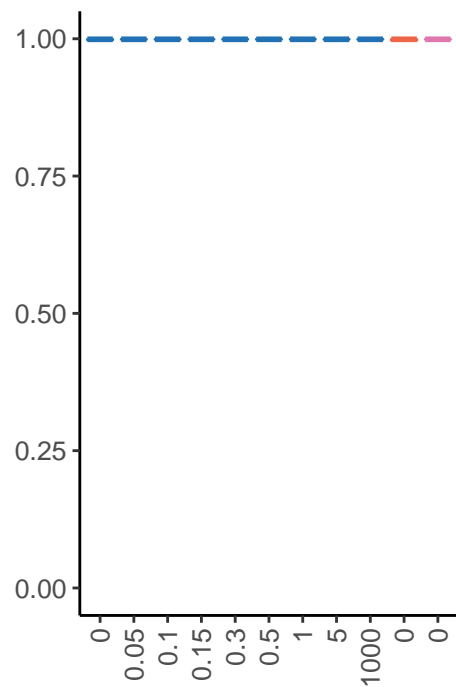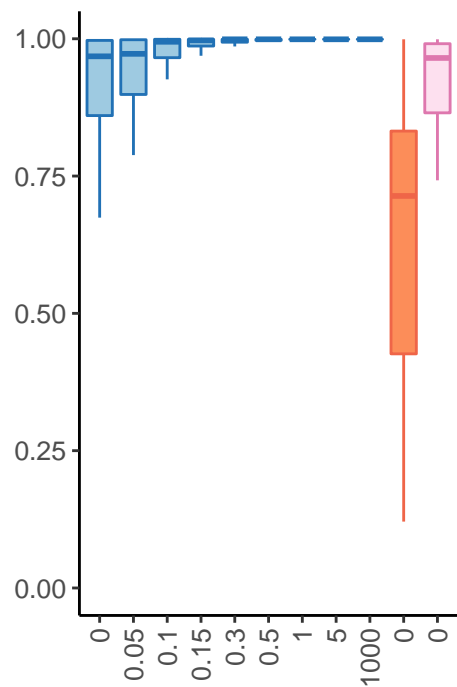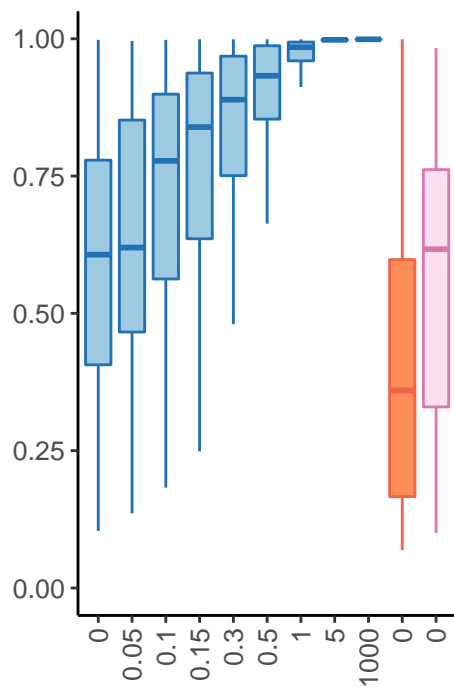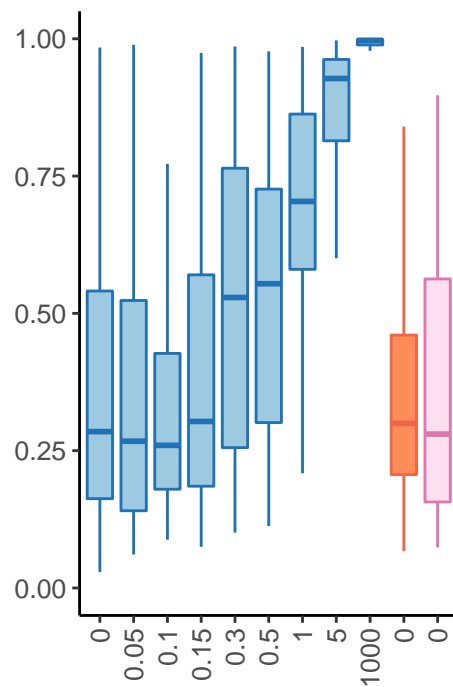

Scenarios

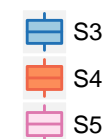

Dispersal rate

Supplement: Supplementary file 1 — Fig. S1. A list of phylogenetic trees of Scenario 2. Fig. S2. A list of phylogenetic trees of Scenario 3. Fig. S3. Parameter estimations for Scenario 2 versus Scenarios 4 and 5. Fig. S4. Parameter estimations for Scenario 3 versus Scenarios 4 and 5. Fig. S5. P‐values and powers of the test of spatial Scenario 2 versus non‐spatial Scenarios 4 and 5. Fig. S6. P‐values and powers of the test of spatial Scenario 3 versus non‐spatial Scenarios 4 and 5. Fig. S7. Local species‐through‐time (STT) plots of Scenario 2 on location 1. Fig. S8. Local species‐through‐time (STT) plots of Scenario 3 on location 1. Fig. S9. Local species‐through‐time (STT) plots of Scenario 3 on location 2. Fig. S10. Nonspatial species‐through‐time (STT) plots of Scenario 1. Fig. S11. Nonspatial species‐through‐time (STT) plots of Scenario 2. Fig. S12. Nonspatial species‐through‐time (STT) plots of Scenario 3. Fig. S13. Lineages‐through‐time (LTT) plots of Scenario 2. Fig. S14. Lineages‐through‐time (LTT) plots of Scenario 3. [file EVO-72-1294-s001.zip › evo13482-sup-0008-PP.S3VS.pdf]

$\mu = 0$  $\mu = 0.1$  $\mu = 0.2$  $\mu = 0.4$  $M_0 = 0$  $M_0 = 0.05$  $M_0 = 0.1$  $M_0 = 0.15$  $M_0 = 0.3$  $M_0 = 0.5$  $M_0 = 1$  $M_0 = 5$  $M_0 = 1000$ 

Number of lineages

Time

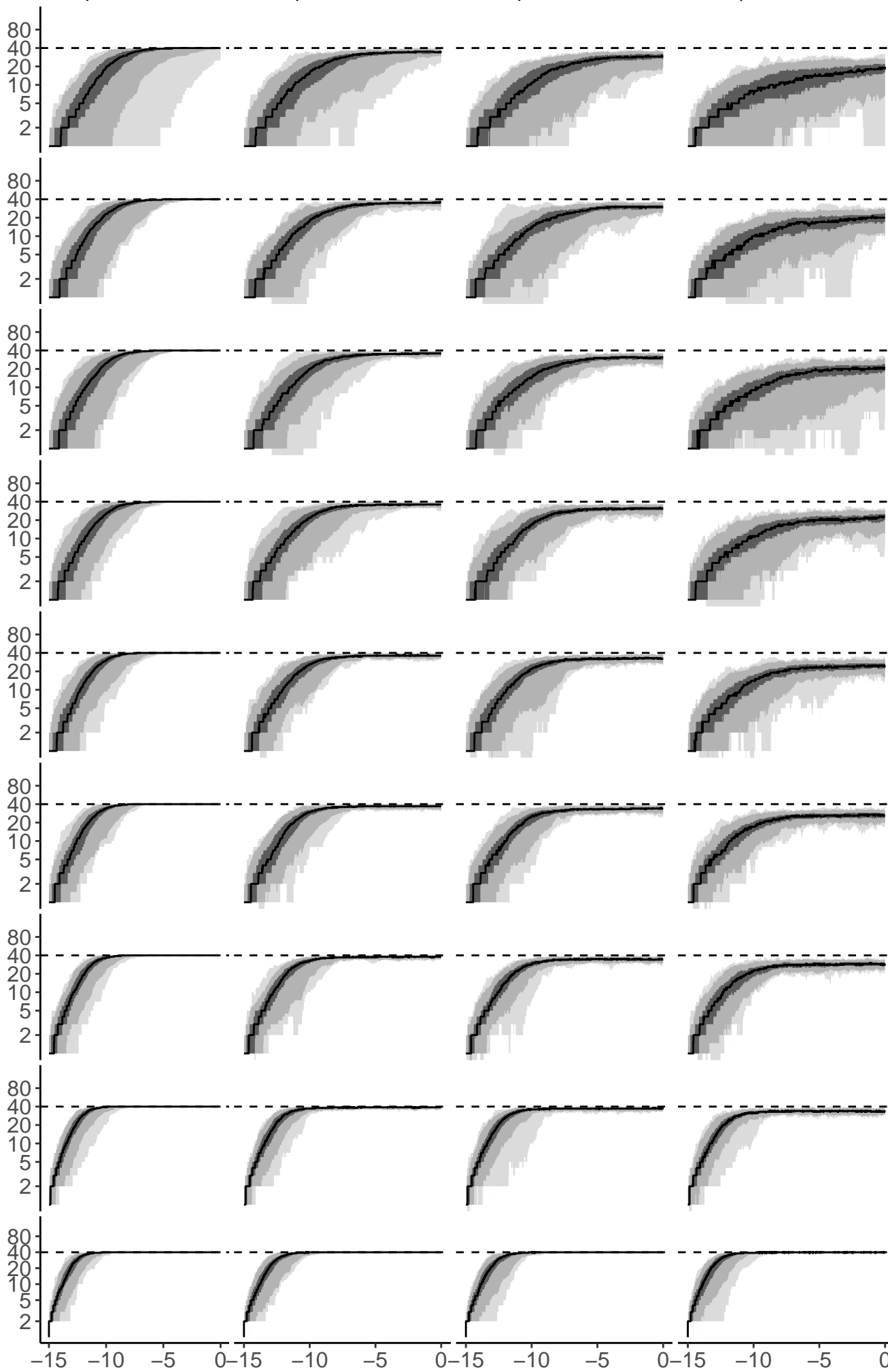

Supplement: Supplementary file 1 — Fig. S1. A list of phylogenetic trees of Scenario 2. Fig. S2. A list of phylogenetic trees of Scenario 3. Fig. S3. Parameter estimations for Scenario 2 versus Scenarios 4 and 5. Fig. S4. Parameter estimations for Scenario 3 versus Scenarios 4 and 5. Fig. S5. P‐values and powers of the test of spatial Scenario 2 versus non‐spatial Scenarios 4 and 5. Fig. S6. P‐values and powers of the test of spatial Scenario 3 versus non‐spatial Scenarios 4 and 5. Fig. S7. Local species‐through‐time (STT) plots of Scenario 2 on location 1. Fig. S8. Local species‐through‐time (STT) plots of Scenario 3 on location 1. Fig. S9. Local species‐through‐time (STT) plots of Scenario 3 on location 2. Fig. S10. Nonspatial species‐through‐time (STT) plots of Scenario 1. Fig. S11. Nonspatial species‐through‐time (STT) plots of Scenario 2. Fig. S12. Nonspatial species‐through‐time (STT) plots of Scenario 3. Fig. S13. Lineages‐through‐time (LTT) plots of Scenario 2. Fig. S14. Lineages‐through‐time (LTT) plots of Scenario 3. [file EVO-72-1294-s001.zip › evo13482-sup-0009-STT_S2L1.pdf]

$\mu = 0$  $\mu = 0.1$  $\mu = 0.2$  $\mu = 0.4$  $M_0 = 0$  $M_0 = 0.05$  $M_0 = 0.1$  $M_0 = 0.15$  $M_0 = 0.3$  $M_0 = 0.5$  $M_0 = 1$  $M_0 = 5$  $M_0 = 1000$ 

Number of lineages

Time

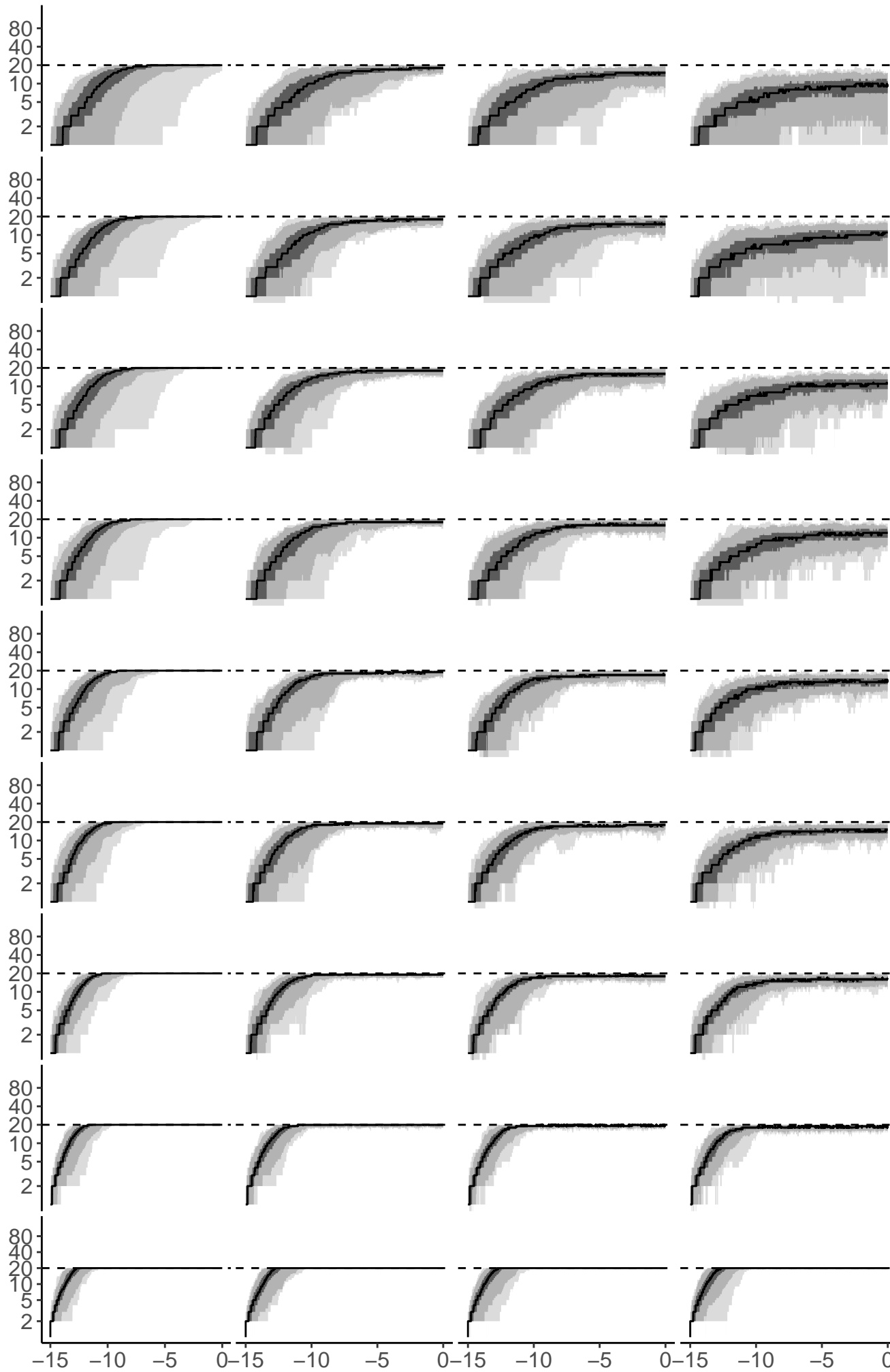

Supplement: Supplementary file 1 — Fig. S1. A list of phylogenetic trees of Scenario 2. Fig. S2. A list of phylogenetic trees of Scenario 3. Fig. S3. Parameter estimations for Scenario 2 versus Scenarios 4 and 5. Fig. S4. Parameter estimations for Scenario 3 versus Scenarios 4 and 5. Fig. S5. P‐values and powers of the test of spatial Scenario 2 versus non‐spatial Scenarios 4 and 5. Fig. S6. P‐values and powers of the test of spatial Scenario 3 versus non‐spatial Scenarios 4 and 5. Fig. S7. Local species‐through‐time (STT) plots of Scenario 2 on location 1. Fig. S8. Local species‐through‐time (STT) plots of Scenario 3 on location 1. Fig. S9. Local species‐through‐time (STT) plots of Scenario 3 on location 2. Fig. S10. Nonspatial species‐through‐time (STT) plots of Scenario 1. Fig. S11. Nonspatial species‐through‐time (STT) plots of Scenario 2. Fig. S12. Nonspatial species‐through‐time (STT) plots of Scenario 3. Fig. S13. Lineages‐through‐time (LTT) plots of Scenario 2. Fig. S14. Lineages‐through‐time (LTT) plots of Scenario 3. [file EVO-72-1294-s001.zip › evo13482-sup-0010-STT_S3L1.pdf]

$\mu = 0$  $\mu = 0.1$  $\mu = 0.2$  $\mu = 0.4$  $M_0 = 0$  $M_0 = 0.05$  $M_0 = 0.1$  $M_0 = 0.15$  $M_0 = 0.3$  $M_0 = 0.5$  $M_0 = 1$  $M_0 = 5$  $M_0 = 1000$ 

Number of lineages

Time

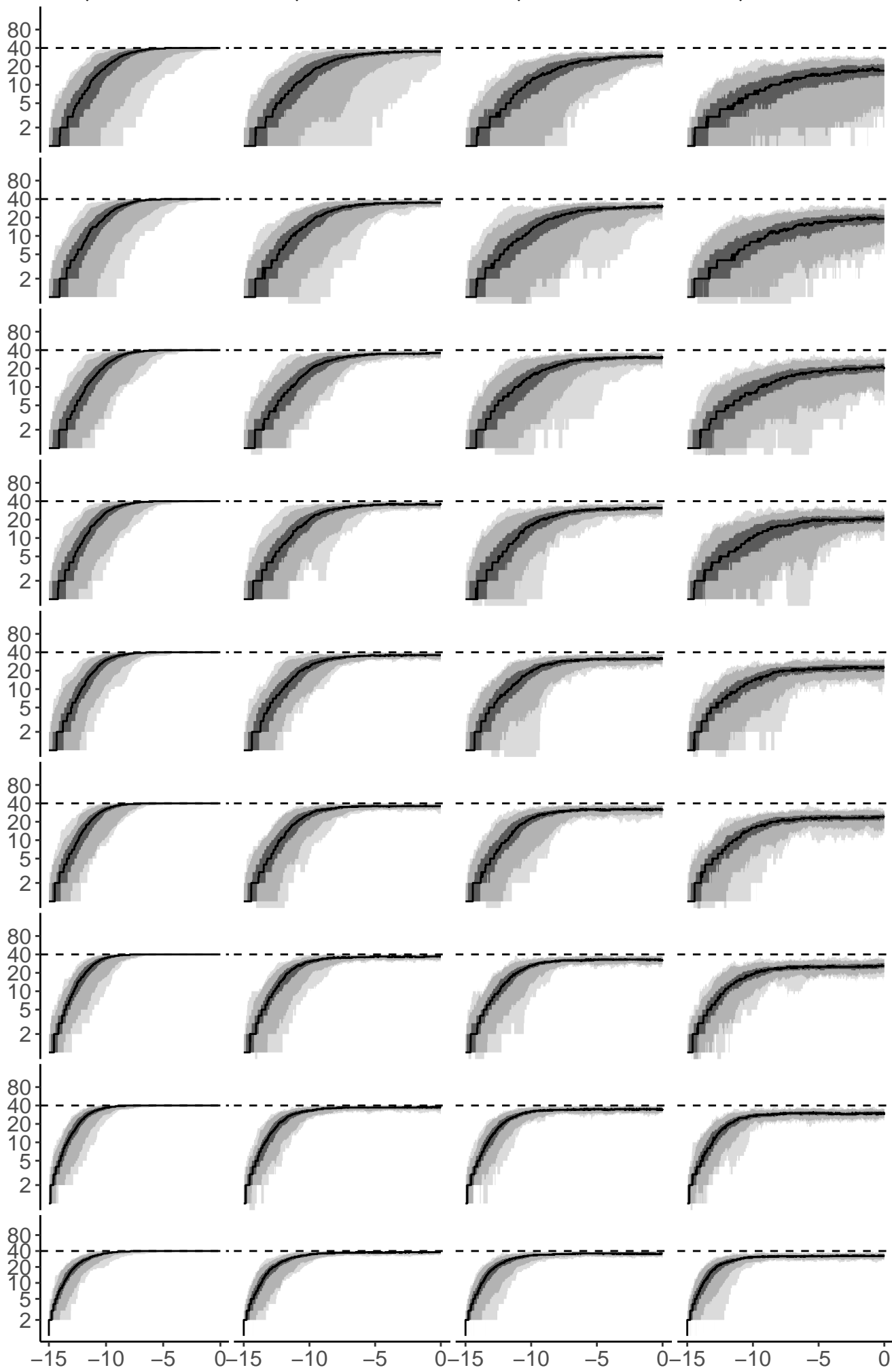

Supplement: Supplementary file 1 — Fig. S1. A list of phylogenetic trees of Scenario 2. Fig. S2. A list of phylogenetic trees of Scenario 3. Fig. S3. Parameter estimations for Scenario 2 versus Scenarios 4 and 5. Fig. S4. Parameter estimations for Scenario 3 versus Scenarios 4 and 5. Fig. S5. P‐values and powers of the test of spatial Scenario 2 versus non‐spatial Scenarios 4 and 5. Fig. S6. P‐values and powers of the test of spatial Scenario 3 versus non‐spatial Scenarios 4 and 5. Fig. S7. Local species‐through‐time (STT) plots of Scenario 2 on location 1. Fig. S8. Local species‐through‐time (STT) plots of Scenario 3 on location 1. Fig. S9. Local species‐through‐time (STT) plots of Scenario 3 on location 2. Fig. S10. Nonspatial species‐through‐time (STT) plots of Scenario 1. Fig. S11. Nonspatial species‐through‐time (STT) plots of Scenario 2. Fig. S12. Nonspatial species‐through‐time (STT) plots of Scenario 3. Fig. S13. Lineages‐through‐time (LTT) plots of Scenario 2. Fig. S14. Lineages‐through‐time (LTT) plots of Scenario 3. [file EVO-72-1294-s001.zip › evo13482-sup-0011-STT_S3L2.pdf]

$\mu = 0$  $\mu = 0.1$  $\mu = 0.2$  $\mu = 0.4$  $M_0 = 0$  $M_0 = 0.05$  $M_0 = 0.1$  $M_0 = 0.15$  $M_0 = 0.3$  $M_0 = 0.5$  $M_0 = 1$  $M_0 = 5$  $M_0 = 1000$ 

Number of lineages

Time

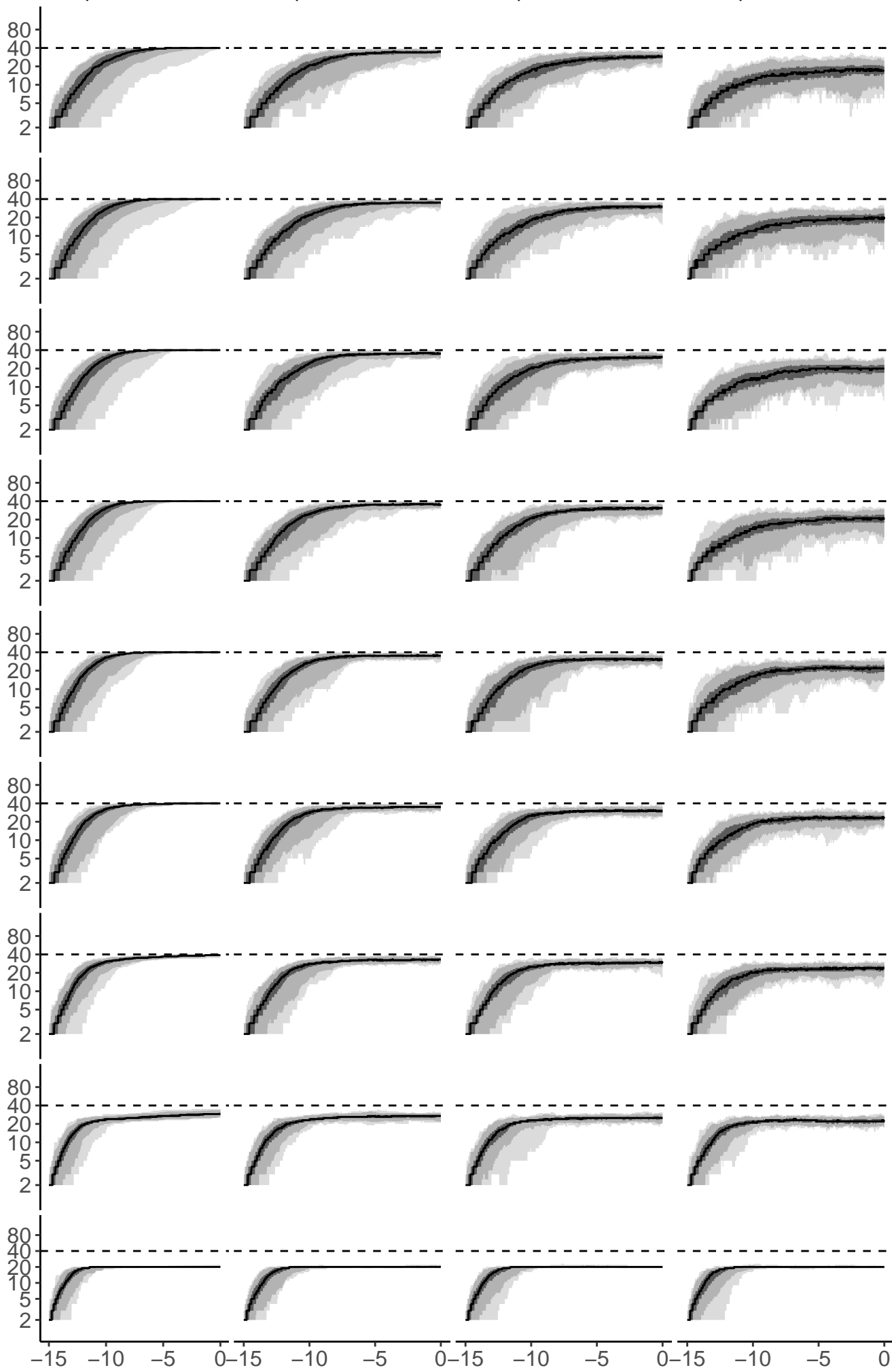

Supplement: Supplementary file 1 — Fig. S1. A list of phylogenetic trees of Scenario 2. Fig. S2. A list of phylogenetic trees of Scenario 3. Fig. S3. Parameter estimations for Scenario 2 versus Scenarios 4 and 5. Fig. S4. Parameter estimations for Scenario 3 versus Scenarios 4 and 5. Fig. S5. P‐values and powers of the test of spatial Scenario 2 versus non‐spatial Scenarios 4 and 5. Fig. S6. P‐values and powers of the test of spatial Scenario 3 versus non‐spatial Scenarios 4 and 5. Fig. S7. Local species‐through‐time (STT) plots of Scenario 2 on location 1. Fig. S8. Local species‐through‐time (STT) plots of Scenario 3 on location 1. Fig. S9. Local species‐through‐time (STT) plots of Scenario 3 on location 2. Fig. S10. Nonspatial species‐through‐time (STT) plots of Scenario 1. Fig. S11. Nonspatial species‐through‐time (STT) plots of Scenario 2. Fig. S12. Nonspatial species‐through‐time (STT) plots of Scenario 3. Fig. S13. Lineages‐through‐time (LTT) plots of Scenario 2. Fig. S14. Lineages‐through‐time (LTT) plots of Scenario 3. [file EVO-72-1294-s001.zip › evo13482-sup-0012-STT_S1L3.pdf]

$\mu = 0$  $\mu = 0.1$  $\mu = 0.2$  $\mu = 0.4$  $M_0 = 0$  $M_0 = 0.05$  $M_0 = 0.1$  $M_0 = 0.15$  $M_0 = 0.3$  $M_0 = 0.5$  $M_0 = 1$  $M_0 = 5$  $M_0 = 1000$ 

Number of lineages

Time

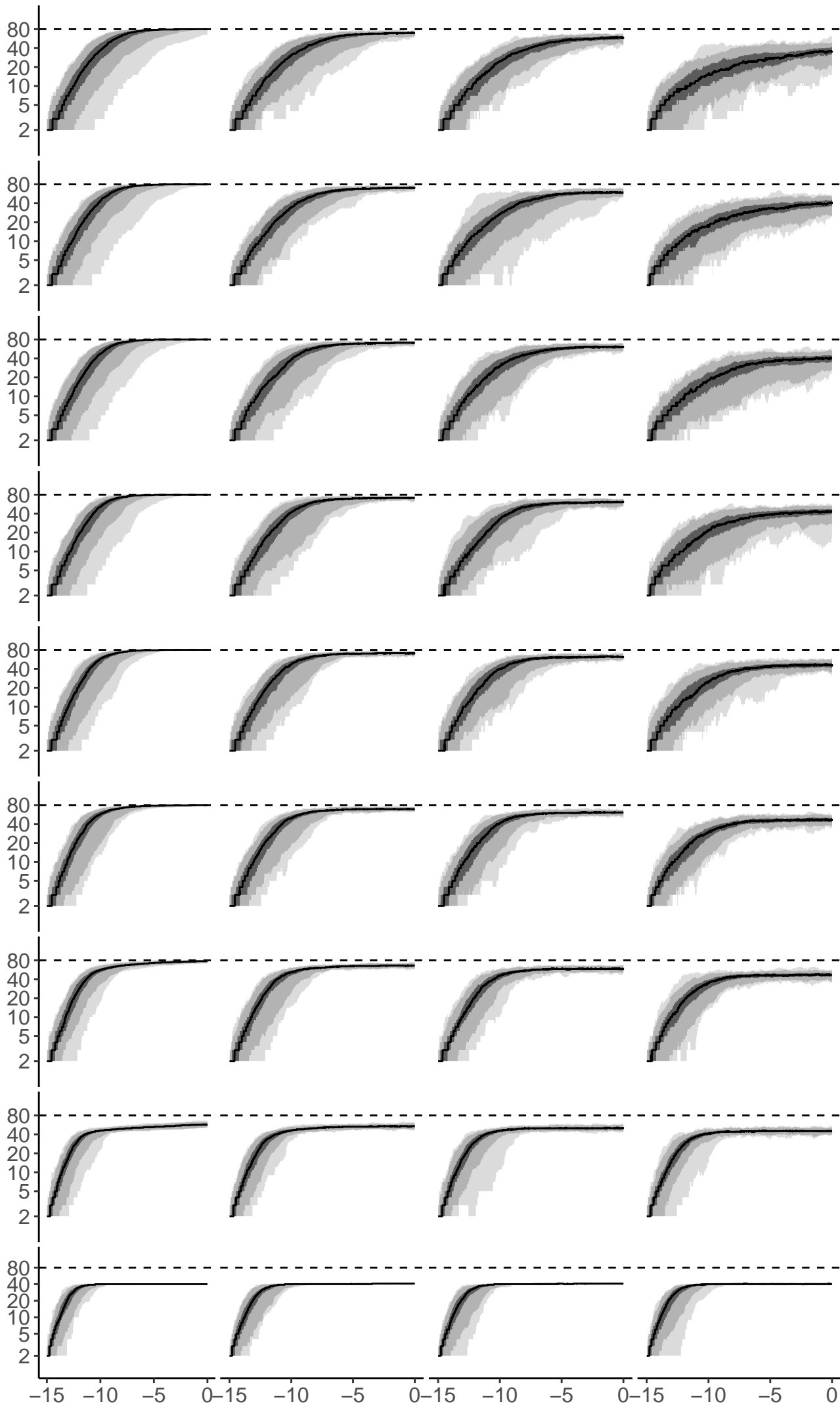

Supplement: Supplementary file 1 — Fig. S1. A list of phylogenetic trees of Scenario 2. Fig. S2. A list of phylogenetic trees of Scenario 3. Fig. S3. Parameter estimations for Scenario 2 versus Scenarios 4 and 5. Fig. S4. Parameter estimations for Scenario 3 versus Scenarios 4 and 5. Fig. S5. P‐values and powers of the test of spatial Scenario 2 versus non‐spatial Scenarios 4 and 5. Fig. S6. P‐values and powers of the test of spatial Scenario 3 versus non‐spatial Scenarios 4 and 5. Fig. S7. Local species‐through‐time (STT) plots of Scenario 2 on location 1. Fig. S8. Local species‐through‐time (STT) plots of Scenario 3 on location 1. Fig. S9. Local species‐through‐time (STT) plots of Scenario 3 on location 2. Fig. S10. Nonspatial species‐through‐time (STT) plots of Scenario 1. Fig. S11. Nonspatial species‐through‐time (STT) plots of Scenario 2. Fig. S12. Nonspatial species‐through‐time (STT) plots of Scenario 3. Fig. S13. Lineages‐through‐time (LTT) plots of Scenario 2. Fig. S14. Lineages‐through‐time (LTT) plots of Scenario 3. [file EVO-72-1294-s001.zip › evo13482-sup-0013-STT_S2L3.pdf]

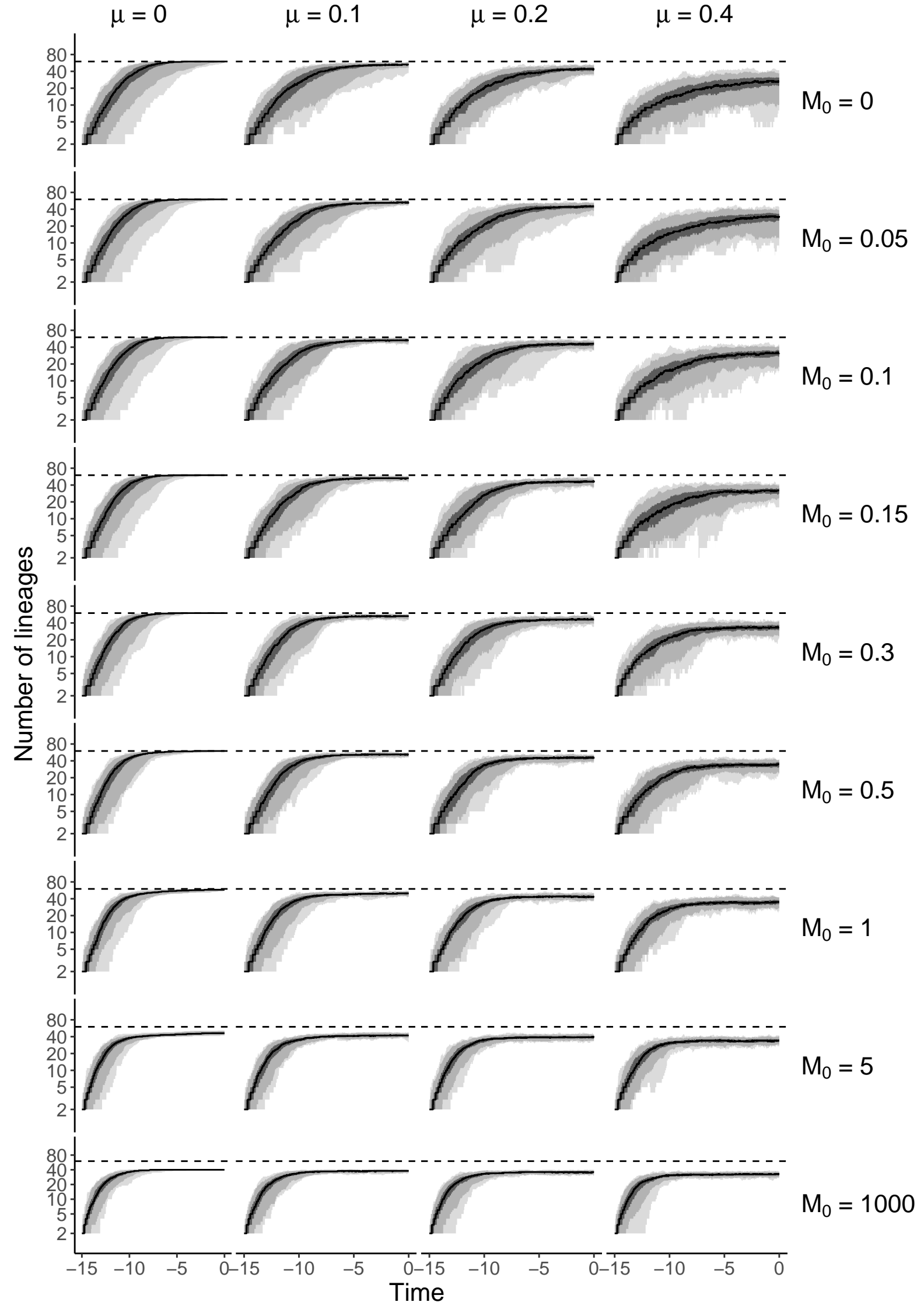

Supplement: Supplementary file 1 — Fig. S1. A list of phylogenetic trees of Scenario 2. Fig. S2. A list of phylogenetic trees of Scenario 3. Fig. S3. Parameter estimations for Scenario 2 versus Scenarios 4 and 5. Fig. S4. Parameter estimations for Scenario 3 versus Scenarios 4 and 5. Fig. S5. P‐values and powers of the test of spatial Scenario 2 versus non‐spatial Scenarios 4 and 5. Fig. S6. P‐values and powers of the test of spatial Scenario 3 versus non‐spatial Scenarios 4 and 5. Fig. S7. Local species‐through‐time (STT) plots of Scenario 2 on location 1. Fig. S8. Local species‐through‐time (STT) plots of Scenario 3 on location 1. Fig. S9. Local species‐through‐time (STT) plots of Scenario 3 on location 2. Fig. S10. Nonspatial species‐through‐time (STT) plots of Scenario 1. Fig. S11. Nonspatial species‐through‐time (STT) plots of Scenario 2. Fig. S12. Nonspatial species‐through‐time (STT) plots of Scenario 3. Fig. S13. Lineages‐through‐time (LTT) plots of Scenario 2. Fig. S14. Lineages‐through‐time (LTT) plots of Scenario 3. [file EVO-72-1294-s001.zip › evo13482-sup-0014-STT_S3L3.pdf]

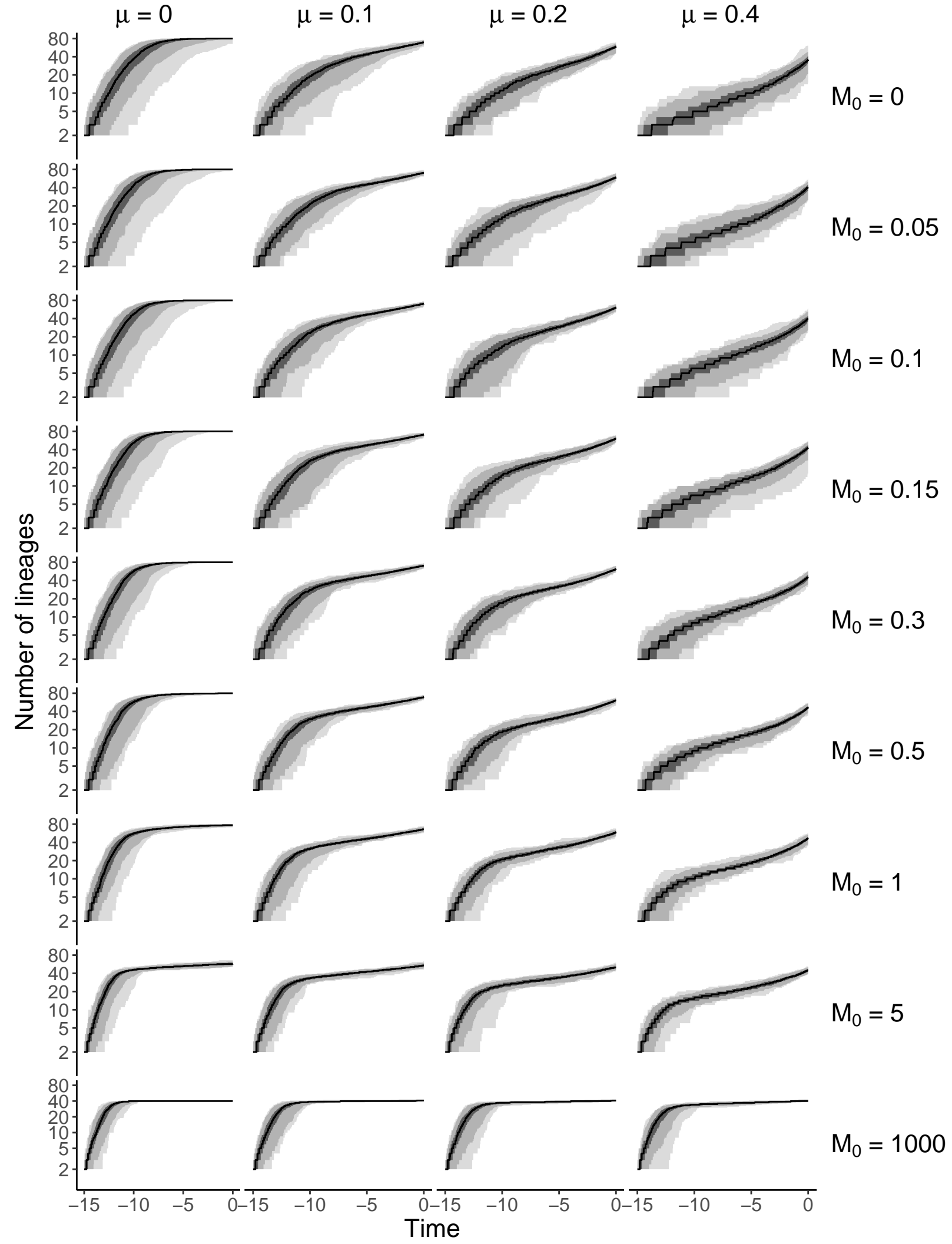

Supplement: Supplementary file 1 — Fig. S1. A list of phylogenetic trees of Scenario 2. Fig. S2. A list of phylogenetic trees of Scenario 3. Fig. S3. Parameter estimations for Scenario 2 versus Scenarios 4 and 5. Fig. S4. Parameter estimations for Scenario 3 versus Scenarios 4 and 5. Fig. S5. P‐values and powers of the test of spatial Scenario 2 versus non‐spatial Scenarios 4 and 5. Fig. S6. P‐values and powers of the test of spatial Scenario 3 versus non‐spatial Scenarios 4 and 5. Fig. S7. Local species‐through‐time (STT) plots of Scenario 2 on location 1. Fig. S8. Local species‐through‐time (STT) plots of Scenario 3 on location 1. Fig. S9. Local species‐through‐time (STT) plots of Scenario 3 on location 2. Fig. S10. Nonspatial species‐through‐time (STT) plots of Scenario 1. Fig. S11. Nonspatial species‐through‐time (STT) plots of Scenario 2. Fig. S12. Nonspatial species‐through‐time (STT) plots of Scenario 3. Fig. S13. Lineages‐through‐time (LTT) plots of Scenario 2. Fig. S14. Lineages‐through‐time (LTT) plots of Scenario 3. [file EVO-72-1294-s001.zip › evo13482-sup-0015-LTT_S2.pdf]

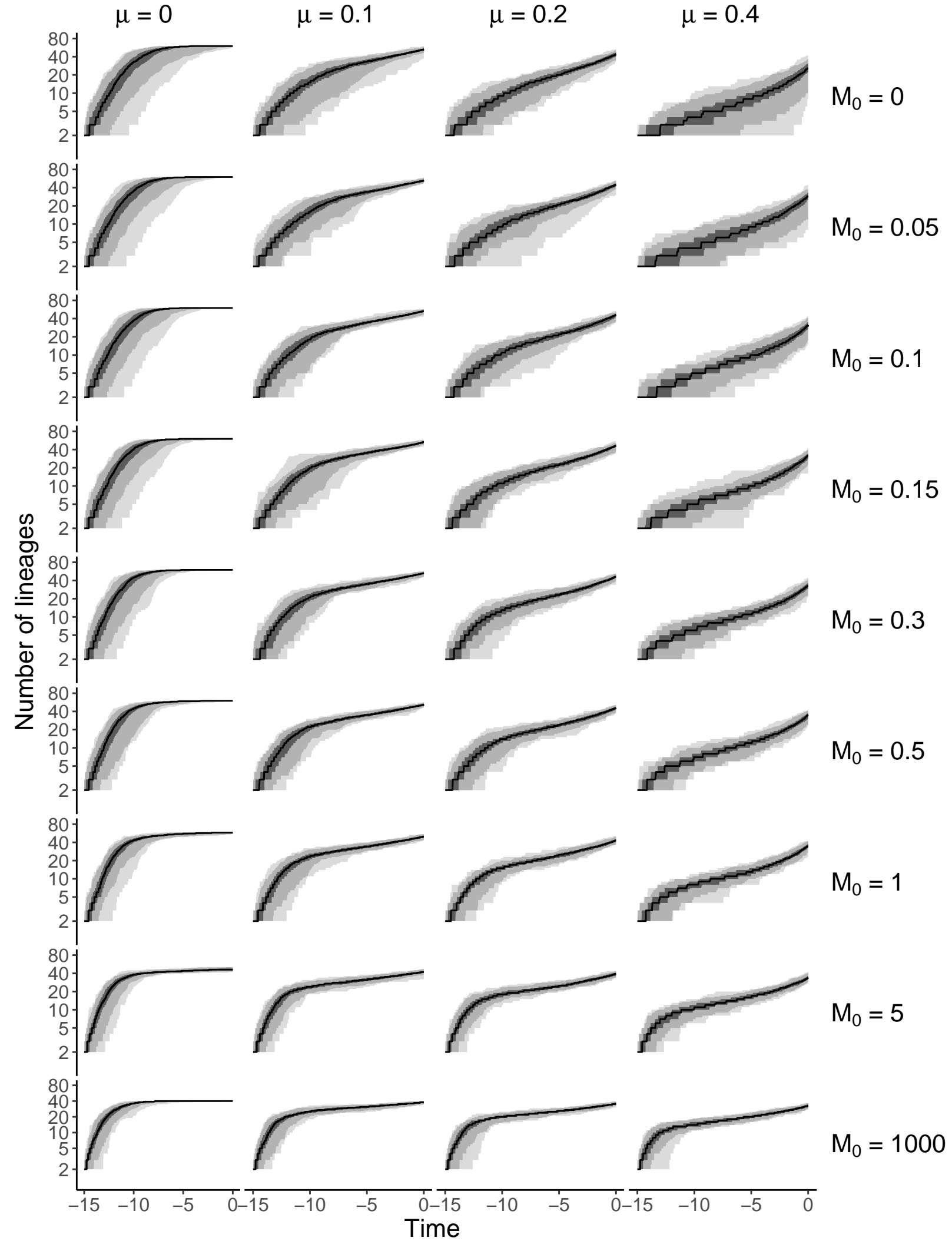

Supplement: Supplementary file 1 — Fig. S1. A list of phylogenetic trees of Scenario 2. Fig. S2. A list of phylogenetic trees of Scenario 3. Fig. S3. Parameter estimations for Scenario 2 versus Scenarios 4 and 5. Fig. S4. Parameter estimations for Scenario 3 versus Scenarios 4 and 5. Fig. S5. P‐values and powers of the test of spatial Scenario 2 versus non‐spatial Scenarios 4 and 5. Fig. S6. P‐values and powers of the test of spatial Scenario 3 versus non‐spatial Scenarios 4 and 5. Fig. S7. Local species‐through‐time (STT) plots of Scenario 2 on location 1. Fig. S8. Local species‐through‐time (STT) plots of Scenario 3 on location 1. Fig. S9. Local species‐through‐time (STT) plots of Scenario 3 on location 2. Fig. S10. Nonspatial species‐through‐time (STT) plots of Scenario 1. Fig. S11. Nonspatial species‐through‐time (STT) plots of Scenario 2. Fig. S12. Nonspatial species‐through‐time (STT) plots of Scenario 3. Fig. S13. Lineages‐through‐time (LTT) plots of Scenario 2. Fig. S14. Lineages‐through‐time (LTT) plots of Scenario 3. [file EVO-72-1294-s001.zip › evo13482-sup-0016-LTT_S3.pdf]
